# Role of Innate lymphoid Cells in Obesity and Insulin Resistance

**DOI:** 10.3389/fendo.2022.855197

**Published:** 2022-04-27

**Authors:** Hong Chen, Lijun Sun, Lu Feng, Yue Yin, Weizhen Zhang

**Affiliations:** ^1^ Department of Physiology and Pathophysiology, School of Basic Medical Sciences, and Key Laboratory of Molecular Cardiovascular Science, Ministry of Education, Peking University, Beijing, China; ^2^ Department of Surgery, University of Michigan Medical Center, Ann Arbor, MI, United States

**Keywords:** innate lymphoid cells, obesity, insulin resistance, immune regulation, metabolic syndrome

## Abstract

Obesity, a growing chronic metabolic disease, greatly increases the risk of metabolic syndrome which includes type 2 diabetes, fatty liver and cardiovascular diseases. Obesity-associated metabolic diseases significantly contribute to mortality and reduce life expectancy. Recently, innate lymphoid cells (ILCs) have emerged as crucial regulators of metabolic homeostasis and tissue inflammation. This review focuses on the roles of ILCs in different metabolic tissues, including adipose tissue, liver, pancreas, and intestine. We briefly outline the relationship between obesity, inflammation, and insulin resistance. We then discuss how ILCs in distinct metabolic organs may function to maintain metabolic homeostasis and contribute to obesity and its associated metabolic diseases. The potential of ILCs as the therapeutic target for obesity and insulin resistance is also addressed.

## 1 Introduction

Obesity is a chronic metabolic disease caused by the excessive accumulation of fat. The global prevalence of obesity is overgrowing. The World Health Organization (WHO) estimates that obese people worldwide have nearly tripled since 1975. From 1975 to 2014, the prevalence of obesity has increased from 3.2% to 10.8% in adult males and from 6.4% to 14.9% in adult females worldwide (1). And the prevalence of obesity among adolescents in the 5-19 years age group has dramatically increased from 1975 to 2016 worldwide. Specifically, the prevalence of obesity has increased from 0.7% to 5.6% in girls and 0.9% to 7.8% in boys (2). Obesity increases all-cause mortality in four continents (3, 4). Metabolic syndrome is a condition characterized by obesity, insulin resistance, hypertension, and hyperlipidemia, which leads to the development of a range of diseases, including type 2 diabetes mellitus, cardiovascular disease, non-alcoholic steatohepatitis and cancer. These metabolic diseases are the leading causes of death nowadays. Obesity thus is a public health and clinical challenge worldwide. Exploring the pathophysiological mechanisms underlying obesity is thus critical for the development of efficient therapeutic strategies to combat this disease.

Insulin resistance, in short, is that insulin cannot function normally. The exact amount of insulin fails to increase the uptake and utilization of glucose in adipose tissue, liver and muscle. The etiology of insulin resistance is recognized as chronic tissue inflammation (5). Several potential mechanisms underlying the development of obesity- associated low-grade inflammation have been proposed. Firstly, obesity increases gut permeability, and gut microbiota-derived substances trigger the inflammation signals by activating receptors such as Toll-like receptor 4 (6). Secondly, obesity elevates various lipids in circulation such as free fatty acids, leading to subsequent activation of TLR2/NFκB pathways (7). Besides, the perturbed phosphocreatine/creatine metabolism in the obese state results in increased transcription of multiple pro-inflammatory genes (8). Thirdly, obesity induces the rapid expansion of adipocytes, which induces adipocyte death, hypoxia and mechanical stress between the cell and the extracellular matrix (ECM), resulting in inflammation (7). Inflammatory signaling such as NFκB and c-Jun N-terminal kinase (JNK) can directly or indirectly block insulin action. For example, JNK phosphorylates insulin receptor substrates at serine/threonine sites rather than the tyrosine site, thereby inhibiting downstream signals of insulin receptors (9). The first evidence that obesity is connected with inflammation is the discovery that TNF-α is overexpressed and promotes insulin resistance in obese mice (10). Later, macrophages and their pro-inflammatory polarization were shown as key risk factors in obesity (11, 12). Subsequently, many other immune cells, such as eosinophils and mast cells, were found to participate in the low-grade chronic inflammation in obesity (13–16). These immune cells orchestrate the local environment of metabolic organs and are connected with insulin resistance in obesity (17–20). Targeting immune pathways in chronic inflammation may successfully prevent or treat obesity and insulin resistance.

Innate lymphoid cells (ILCs) are a recently identified group of innate lymphocytes which lack antigen-specific receptors expressed on T and B lymphocytes (21). On the basis of developmental pathways, the ILC family have been divided into five subsets: natural killer (NK) cells, group 1 ILCs (ILC1s), group 2 ILCs (ILC2s), lymphoid tissue inducer (LTi) cells, and group 3 ILCs (ILC3s) (22). They are considered as the innate counterparts of T lymphocytes, which have been introduced in many reviews (23–25). ILCs reside in the intestine, lung, adipose tissue, liver, and pancreas, and react rapidly to environmental stimuli (26). Mature ILCs are activated by cytokines, alarmins, and inflammatory mediators from myeloid cells or epithelial cells. For example, NK cells express a range of NK cell receptors (NKRs) which recognize numerous ligands on target stressed cells (27–29). IL-12 activates ILC1s, while ILC2s are stimulated by IL-33 and IL-25 (30). IL-33 induces strong activation of ILC2s through the receptor suppression of tumorigenicity 2 (ST2) (31, 32). RORγt^+^ ILC3s are activated by IL-23 and then produce IL-17 and IL-22 (33, 34). They quickly respond to stress signals and maintain tissue homeostasis. However, they may also participate in the progression of inflammation. Recently, studies have unveiled the role of ILCs in metabolism. The functions of ILCs in different metabolic tissues are being actively investigated in depth as a link between the immune system and the metabolic system.

Obesity-induced chronic low-grade inflammation occurs in multiple metabolic organs, including adipose tissue, liver, pancreas, and intestine. Inflammation can lead to tissue damage, necrosis, and fibrosis. ILCs in these metabolic organs function to maintain homeostasis or contribute to inflammation. Here, we focus on the roles of ILCs in obesity and insulin resistance, discuss how ILCs in different tissues regulate metabolic homeostasis to protect against obesity or how they contribute to inflammation and insulin resistance. Targeting ILCs and their associated immune pathways may represent a novel approach to treat obesity and insulin resistance.

## 2 ILCs in Adipose Tissue

Adipose tissue is a dynamic organ regulating the homeostasis of energy (35). When energy intake exceeds energy expenditure, excess energy stores in white adipose tissue (WAT) in the form of triglycerides. In normal circumstances, insulin activates lipoprotein lipase and inhibits hormone-sensitive lipase and thus increases absorption and deposition of triglyceride in the adipose tissue after food intake. However, excessive fat accumulation leads to adipocytes hypertrophy and hyperplasia which results in inflammation and insulin resistance in the WAT (36). In contrast to the white adipocytes whose main function is storing triglyceride, beige adipocytes are thermogenic cells that can promote energy consumption (37). Beige adipocytes are inducible and plastic. When exposed to cold stimulation or β3-adrenergic receptor agonists, the white adipose tissue can expend energy by increasing the number of beige adipocytes and improving their activity. Beige adipocytes exist in WAT and are differentiated from Myf5 negative adipose progenitor cells or transformed from mature white adipocytes. They can increase the body’s energy expenditure and improve glucose and lipid metabolism, thus becoming promising targets for preventing and treating obesity and insulin resistance. Recently, Trim et al. have reviewed that leukocytes in the adipose tissue regulate the homeostasis of adipocytes and respond to the changes of nutrition and body temperature (38). Here, we focus on the function of ILCs in the adipose tissue in both health and obese associated metabolic disease.

### 2.1 NK Cells and ILC1s Regulate the Inflammation in Adipose Tissue

**Figure 1:**
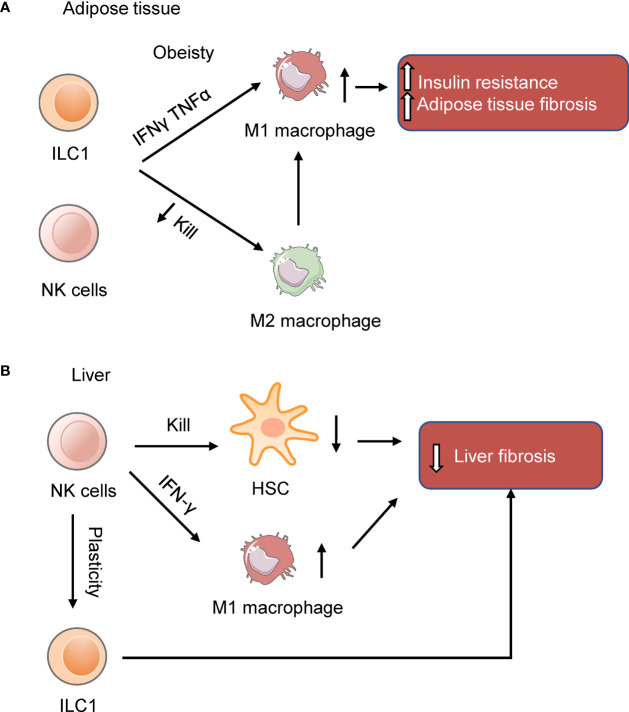
NK cells and ILC1s in the adipose tissue and liver. **(A)** NK cells and ILC1s produce IFNγ and TNFα to induce insulin resistance in obesity by inducing M1 macrophages and adipose tissue fibrosis. **(B)** NK cells in the liver prevent liver fibrosis by killing hepatic stellate cells (HSC) or inducing macrophages M1 polarization. In the obese liver, NK cells are more like ILC1s (39). The reduction of NK cell cytotoxicity may benefit the liver in NAFLD.

Recent studies showed that NK cells and ILC1s in the adipose tissue participate in developing inflammation and insulin resistance in obese mice (**Figure 1A**). Diet-induced obesity (DIO) increases NK cell number and induces NK cells to produce IFN-γ and TNFα in the visceral adipose tissue (VAT). IFN-γ and TNFα induce type 1 macrophages (M1 macrophages) accumulation and promote insulin resistance. Ablation of NK cells prevents the differentiation of M1 macrophages, reduces inflammation, and restores insulin sensitivity (**Table 1**) (40–42), while expansion of NK cells exacerbates DIO-induced inflammation and insulin resistance (41). Similarly, IL-12 acts on IL-12R, activating STAT4, and then induces production of IFN-γ from ILC1s in DIO mice, resulting in the expansion of M1 macrophages and insulin resistance (43). Other than secreting cytokines, group 1 ILCs constrain macrophages through cytotoxicity as macrophages express stress ligand of activating receptor NKG2D. The killing ability of ILC1s is impaired in DIO mice, changing the proportion of M1 macrophages and anti-inflammatory M2 macrophages, leading to subsequent metabolic disorders (44). Furthermore, a recent study shows that obesity increases the number of a specific interleukin-6 receptor (IL6R) a^+^ NK subpopulation in mice and humans. This specific NK cell population facilitates obesity and insulin resistance (45). The exact site of origin, the precursors and the factors to stimulate IL6Ra^+^ NK cells, remain unclear. Nevertheless, these results show that NK cells and ILC1s contribute to obesity and insulin resistance.

**Table 1:** Summary of ILCs depletion strategies.

Animal genotype	Ablation of cells
*Rag2^-/-^* mice	T cells, B cells
*Il2rg^-/-^ Rag2^-/-^* mice	ILCs, T cells, B cells
*Il15^-/-^ Rag2^-/-^* mice	ILC1s, NK cells, ILC3s, T cells, B cells
Cre-Ert2 Tg (B6.129 Gt(ROSA)26Sortm1(cre/ERT2)Tyj/J) × Gata3^flox/flox^ mice + 4-hydroxytamoxifen	ILC2s, Th2 cells
*Rorc^gfp/gfp^*	ILC3s, LTi cells, Th17 cells
NKp46-Cre × loxP-stop codon-loxP huDTR + diphtheria toxin (DT)	NK cells, ILC1s, NKp46^+^NKT cells and NKp46^+^ILC3s

Human adipose tissue-resident ILC1s include two subsets, CD56^+^CD127^lo^ ILC1^-^like population and CD56^dim^ CD16^+^ peripheral NK-like subset cells (44). Increased number of adipose and circulating ILC1s has been detected in obese type 2 diabetes patients. Patients with higher levels of ILC1s are associated with a greater risk of type 2 diabetes (46). Bariatric surgery decreases circulating ILC1s numbers and improves metabolic disorders. Adipose tissue ILC1s of type 2 diabetes patients promote adipose fibrogenesis and CD11c^+^ macrophage activation (47). Besides, IFNγ^+^ NK cells play a role in the progression of human obesity. IFNγ^+^ NK cells are positively correlated with inflammation in adipose tissue, plasma glucose levels, and insulin resistance (48). These data show that ILC1s in human adipose tissue promote adipose inflammation and fibrosis in obesity-related type 2 diabetes.

NK cells in obese people are activated, stressed and fail to proliferate or lyse tumors (49, 50). Obesity makes robust lipid accumulation in NK cells, inhibiting their mTOR signaling, blocking their cytotoxic effector functions (51). Interestingly, physical exercise and caloric restriction can increase NK cell cytotoxicity in mice (52, 53). These results collectively demonstrate that the obese environment impairs peripheral NK cells functions and suggest that metabolic reprogramming of NK cells may impair immune cell function, increasing the risk of obesity-related diseases.

Overall, NK cells and ILC1s contribute to insulin resistance in obesity by induction of M1 macrophages and adipose tissue fibrosis. Targeting the pathways of NK cells and ILC1s in adipose tissue may provide new strategies for treating obesity and associated disease.

### 2.2 ILC2s Regulate Metabolic Homeostasis in the Adipose Tissue

**Figure 2:**
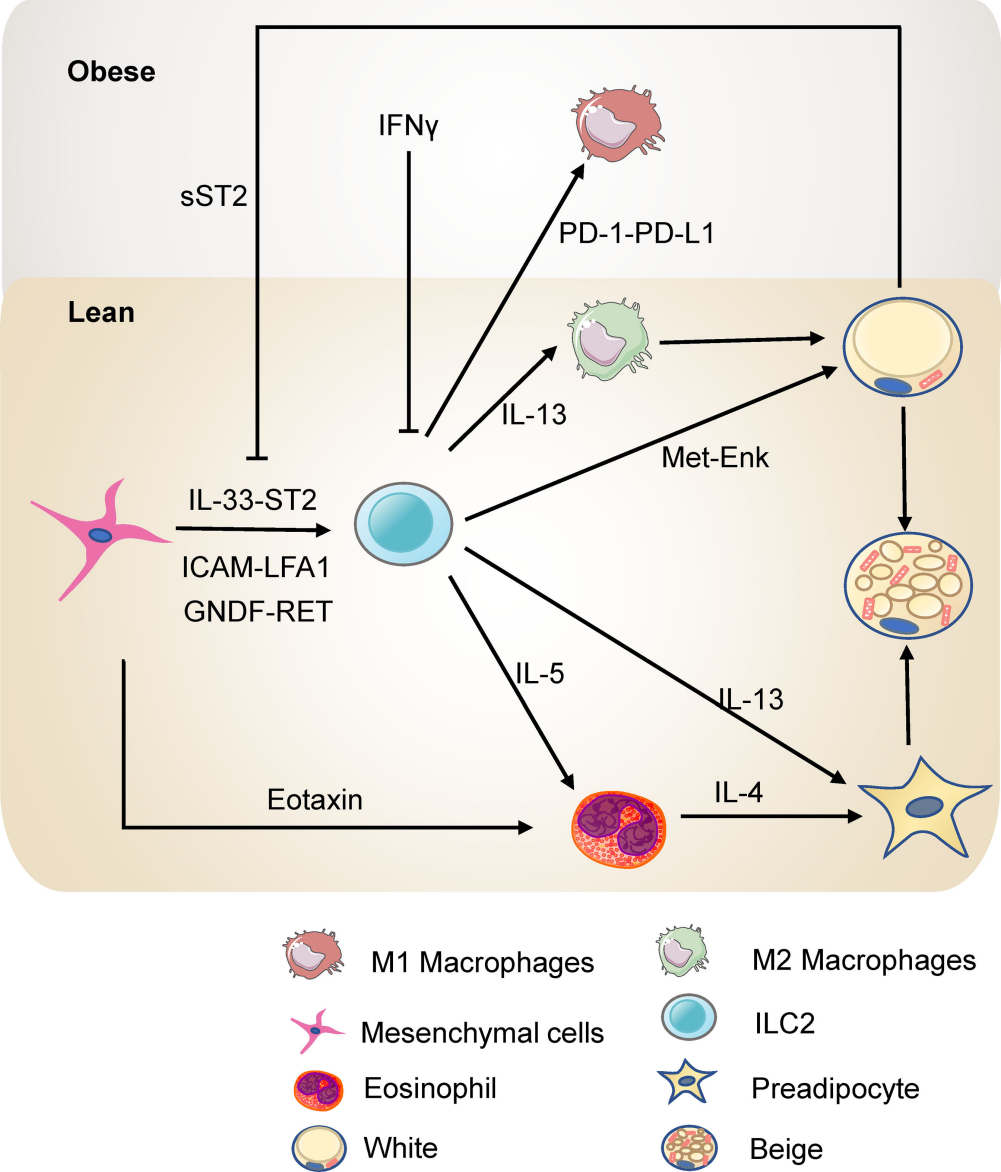
ILC2s in the adipose tissue in lean and obesity state. ILC2s promote the accumulation of eosinophils and M2 macrophages through IL-5 or IL-13 and thus protect against insulin resistance. ILC2s can also directly promote the beigeing of subcutaneous WAT. ILC2s are regulated by the mesenchymal cells and adipocytes in the adipose tissue directly or indirectly.

ILC2s limit obesity and insulin resistance and control the metabolic homeostasis in adipose tissue (**Figure 2**). ILC2s in adipose tissue increase the number of eosinophils and M2 macrophages by type 2 cytokines IL-5 or IL-13 (54). Eosinophils in adipose tissue maintain M2 macrophages and thus promote insulin sensitivity and metabolic homeostasis (55, 56). The deficiency of IL-5 significantly reduces visceral adipose tissue (VAT) eosinophils, increasing obesity and insulin resistance in high fat diet (HFD) fed mice (54). Further, infiltration of ILC2s into VAT by IL-25 administration leads to weight loss and improves glucose tolerance in obese mice. Consistently, transferring ILC2s into obese mice also shows that ILC2s prevent diet-induced obesity (57). In addition, engagement of glucocorticoid-induced tumor necrosis factor receptor (GITR) on activated ILC2s with GITR agonist, DTA-1, induces type 2 cytokines by ILC2s. Experiments of *Rag2* deficient mice injected with DTA-1 and adoptive transfer of adipose ILC2s to GITR^-/-^ mice injected with DTA-1 shows that engagement of GITR on ILC2s is protective against insulin resistance. Further, transfer experiment of IL5^-/-^ or IL-13^-/-^ ILC2s shows that the protective effects of GITR engagement depends on IL-13 particularly (58). Moreover, ILC2s are present in para-aortic adipose tissue. Diet-induced obesity reduced the number of ILC2s in para-aortic adipose tissue. Expansion of ILC2s improves the progression of atherosclerosis while ablation of ILC2s exacerbates atherosclerosis. Bone marrow transplantation experiments showed that the function of ILC2s on atherosclerosis is dependent on IL-5 and IL-13 (59). Thus, ILC2s regulate metabolic homeostasis partly through type 2 cytokines.

ILC2s in white adipose tissue (WAT) contribute to thermogenesis. Strikingly, ILC2s activated by interleukin-33 (IL-33) are sufficient to promote WAT beigeing in thermoneutral mice. ILC2s secrete IL-13 which targets IL-4R in PDGFRα+ adipose precursor cells and promotes beige adipogenesis (60). This research highlights the critical role of ILC2s and type 2 cytokines in regulating adipose precursor cell number and fate. Another study shows that ILC2s are present in human WAT and demonstrates that ILC2s in WAT are dysregulated in obesity. Notably, this study provides a novel mechanism by which IL-33-induced ILC2s drive white fat beigeing. It is not dependent on the eosinophil/IL-4Rα/macrophage pathway or the adaptive immune system. Instead, ILC2s express proprotein convertase subtilisin/kexin type 1 (Pcsk1) which processes the production of methionine-enkephalin (Met-Enk), which directly acts on adipocytes and promotes beige adipocyte formation (61). In addition, cold exposure elevates the level of IL-33, ILC2s, and eosinophils in subcutaneous adipose tissue. Blocking the IL-33 signal reverses the expression of the thermogenic gene UCP1, highlighting that ILC2s are involved in cold-induced thermogenesis (62). Interestingly, a recent study has reported that loss of ILC2s in adipose tissue drives thermogenic failure in aging. ILC2s are lost in aging, and an adoptive transfer experiment showed that adult ILC2s could help old mice resist cold (63). These studies shed light on the role of ILC2s in regulating metabolism and may represent a novel approach for treating obesity.

### 2.3 ILC2s Interact With Local Cells in the Adipose Tissue

As tissue-resident cells, ILC2s interact with the stromal cells and adipocytes in adipose tissue to regulate metabolic homeostasis. White adipose tissue pluripotent mesenchymal cells produce IL-33, increasing the proliferation of ILC2s and the production of type 2 cytokine, thus promoting regulatory circuits that maintain WAT homeostasis (64, 65). Studies by Shan et al. have further demonstrated (66) that IL-33 is only expressed in DPP4^+^ cells and its expression is directly regulated by the β1- adrenergic receptor signaling pathway and CREB protein. Cold exposure rapidly stimulates DPP4^+^ cells to secrete IL-33, which in turn induces the proliferation and activation of ILC2s, thereby promoting white adipose beigeing. IL-33 increases death receptor 3 (DR3) expression on ILC2s and activates the NF-κB pathways, thus stimulating ILC2s and protecting against insulin resistance (67). Besides, activation of ILC2 by IL-33 increases the expression of PPARγ, which is indispensable for the proliferation and expression of cytokines of ILC2s. Inhibition of PPARγ decreases expression of CD36 and uptake of fatty acids (68). IL-33 increases the uptake of lipids and glucose of ILC2s to promote the proliferation of ILC2s in the context of allergen-driven airway inflammation (69). On the other hand, sST2, the soluble isoform of the IL-33 receptor ST2, secreted by adipocytes, attenuates the signaling of IL-33 and disrupts the ILC2 homeostasis in adipose tissue, thereby exacerbating obesity-associated insulin resistance. Zbtb7b, a negative regulator of adipocyte expression of sST2, maintains glucose homeostasis and prevents insulin resistance at a steady-state (70). In addition, the deficiency of ST2 decreases ILC2s in WAT, resulting in increased visceral fat, decreased browning, and impairment of glucose metabolism (71). Meanwhile, adipokine Chemerin and its receptor chemokine-like receptor 1 (CMKLR1) inhibit adipocyte cAMP-PKA signaling, interfering with cold-induced IL-33 secretion and downstream ILC2 activation. This action thereby inhibits white adipose tissue beigeing, leading to obesity and metabolic disorders (72). Other than IL-33, pluripotent mesenchymal cells express the intercellular adhesion molecule ICAM-1, while ILC2s express its ligand LFA antigen 1 (LFA-1). This direct interaction also promotes ILC2s and induces their production of cytokines, which induces mesenchymal cells to secrete eotaxin and support eosinophil recruitment (73). Besides, fibroblasts in adipose tissue express a classical cadherin, cadherin-11, that mediates cell-to-cell adhesion. In Cadherin-11-deficient mice, the stromal cells produced more IL-33 which increased the activity of adipose tissue ILC2s and M2 macrophages, thus reducing inflammation, fibrosis, and glucose intolerance (74). Moreover, adipose mesenchymal cells express glial-derived neurotrophic factor (GNDF) upon stimulation by sympathetic nerve terminals through the β2-adrenergic receptor. GNDF regulates adipose tissue-resident ILC2s, ameliorating high-fat diet-induced obesity (75). Although murine intestinal ILC2s express the β2-adrenergic receptor (β2-AR), which negatively regulates ILC2s responses (76), the adipose ILC2s are mainly regulated indirectly by the sympathetic signals through mesenchymal cells. These studies reveal that mesenchymal cells and adipocytes have a multifaceted dialogue with ILC2s to maintain type 2 immune microenvironment in white adipose tissue.

ILC2s also interact with other immune cells through newly identified pathways in the adipose tissue. After IL-33 treatment, ILC2s interact with T cells *via* ICOSL-ICOS, promoting Treg cell accumulation. On the other hand, IFN-γ treatment inhibits ILC2 activation and reduces the interaction of ILC2s and T cells, thus reducing Treg cell accumulation. Interestingly, this repression increases with HFD-induced obesity (77). Besides, ILC2s express OX40 ligand (OX40L), which interacts with OX40 on T cells to sustain Treg cells and Th2 cells responses in adipose tissue after IL-33 induction (78). Thus, ILC2s mediate type 2 immune responses, sustaining metabolic homeostasis in a lean state. In obesity, TNF triggers IL-33-dependent expression of PD-1 on ILC2s and further recruits and activates PD-L1^hi^ M1 macrophages. PD-1-PD-L1 pathway is responsible for ILC2 destabilization after HFD and results in impaired metabolism in obesity (79). Besides, a hybrid cytokine IL233 with the activities of both IL-2 and IL-33 protects mice from obesity-linked diabetic nephropathy with a more significant accumulation of Tregs, ILC2s, M2 macrophages, and eosinophils in VAT (80). This evidence reveals the crosstalk between ILC2s and other immune cells in adipose tissue, providing novel targets to ameliorate obesity.

### 2.4 ILC3s Are Related to Obesity in Human Adipose Tissue

The function of ILC3s in adipose tissue is less studied. O’Sullivan has reported that ILC3s are absent in lean or obese mouse white adipose tissue (43). Consistently, Sasaki et al. have reported that adoptive transfer of bone marrow cells from *Rag2^−/^* ^−^ mice into *Il2rg^−/−^Rag2^−/−^* mice fails to increase ILC3s characterized as lineage^-^KLRG1^−^IL-7Rα^+^Thy-1^+^ cells in the adipose tissue (81). However, studies using single cell RNA-seq and flow cytometry by Hildreth et al. have recently demonstrated the presence of ILC3s in human white adipose tissue (82). Importantly, the frequency and density of ILC3s increases in obese white adipose tissue compared with healthy people. The frequency and density of ILC3s are positively correlated with patient BMI. What’s more, they have identified a group of ILC precursor (ILCP) cells in human adipose tissue which give rise to ILC1s and ILC3s, but not ILC2s. Further studies are needed to verify whether ILC3s regulate the metabolic homeostasis in the lean state or contribute to the inflammation by expressing LIF, TNFSF13B and MIF in the obese state. The distinct function for ILC3s in adipose tissue between mice and human being suggests that ILCs may not be evolutionarily conserved.

## 3 ILCs in Liver

Non-alcoholic fatty liver disease (NAFLD) is a growing chronic liver disease worldwide, which can lead to cirrhosis and even hepatocellular carcinoma (83, 84). The global prevalence of NAFLD is currently about 25%. Assessing the prevalence of NAFLD in different geographic regions revealed that NAFLD is prevalent on all continents, with South America (31%) and the Middle East (32%) having the highest prevalence, followed by Asia (27%), North America (24%), and Europe (23%), and Africa the lowest prevalence (13%) (85). The progression of NAFLD is closely related to insulin resistance and metabolic syndrome (86). With the increasing prevalence of obesity, type 2 diabetes, and metabolic syndrome, NAFLD is expected to become the leading cause of cirrhosis requiring liver transplantation in the next decade (87). NAFLD includes a range of liver lesions, including simple steatosis, steatohepatitis (Non-alcoholic steatohepatitis, NASH), and fibrosis. During the progression of NAFLD, innate immune cells play a significant role (88).

### 3.1 NK Cells and ILC1s Regulate the Progression of NAFLD

NK cells account for 30–50% of the total lymphocytes in the human liver (89). They are important during the progression of NAFLD. Here we introduce the related studies of NK cells in different stages of NAFLD.

In the stage of HFD-induced liver steatosis, NK cells produce osteopontin, which induces hepatic ER stress and promotes insulin resistance. Ablation of NK cells with neutralizing antibody can improve HFD-induced ER stress, insulin resistance, and liver steatosis (90). This study shows a pathogenic role of NK cells. On contrary, in the liver of obese mice fed 24 weeks on a high fat and sugar diet, NK cells are less cytotoxic, more like ILC1s, and seem to be protective against NAFLD, although the reduction of cytotoxicity increases the susceptibility to cancer. This shift of liver NK cells to ILC1s reflect the plasticity of NK cells. Reducing the cytotoxicity by perforin knockout alleviates the symptoms of NAFLD in mice (39).

Besides, NK cells prevent NASH progression to fibrosis by regulating liver macrophages polarization. In the NASH model of mice fed with a methionine and choline deficient (MCD) diet, DX5^+^NKp46^+^ NK cells increased, which induced macrophages M1-polarized through the production of IFN‐γ by NK cells. Accordingly, ablation of NKp46^+^ cells makes macrophage shift toward M2 phenotypes, which fail to clear damaged cells effectively, thereby promoting the development of fibrosis (91). Besides, genetic deletion of TNF-related apoptosis-inducing ligand (TRAIL) receptor reduces inflammatory macrophages in the liver and suppresses steatohepatitis in FFC (a diet high in saturated fat, cholesterol, and fructose)-fed mice (92). As the mice used in this study is whole body knockout of TRAILR, the reduction in hepatocyte lipoapoptosis may occur after the improved metabolic niche. And this research can’t identify the tissue-specific roles of TRAIL signaling. Using tissue- and cell-specific TRAILR^-/-^ mice may address these problems. Despite the changes of NK cells in mouse model, in patients with NAFLD confirmed by biopsy, the number and function of NK cells is not altered, except for the increased expression of NKG2D on NK cells in NASH patients (93). Further studies exploring how NASH affects NK cells in humans is needed.

Many studies reported the protective role of NK cells in liver fibrosis induced by carbon-tetrachloride (CCl_4_). Melhem et al. reported that NK cells can improve liver fibrosis by killing activated hepatic stellate cells (HSC) (94). HSC are dominant contributors to liver fibrosis and give rise to 82-96% of myofibroblasts (95, 96). Radaeva et al. employed the fibrosis model of mice fed with the 3,5-diethoxycarbonyl-1,4-dihydrocollidine (DDC) diet or injected with CCl_4_. They further found that NK cells kill activated HSC dependent on retinoic acid early inducible 1/NKG2D and TRAIL. NK cells tend to lyse the activated HSC as the activated HSC rather than the quiescent HSC express the NKG2D ligand (97). Other than ligand for NKG2D, murine and human HSC express the ligand for NKp46 of NK cells. NK cells kill HSC dependent on NKp46, and thus ameliorating liver fibrosis induced by CCl_4_ (98). Besides, IL-18 and TLR3 ligand activated NK cells kill HSC through the p38/PI3K/AKT-dependent pathway *in vitro (* 99). These studies revealed that NK cells protect against the liver fibrosis by killing HSC.

Besides, NK cells are involved in the development of hepatocellular carcinoma (100). NK cells are important for the surveillance of hepatocellular carcinoma. In patients with hepatocellular carcinoma, the number of NK cells significantly decreased (101). NK cells are regulated by monocytes and macrophages by CD48/2B4 axis in hepatocellular carcinoma (102). Besides, myeloid derived suppressor cells inhibit the cytotoxicity and production of cytokines from natural killer cells *via* the NKp30 receptor in hepatocellular carcinoma (103). Moreover, fibroblasts inducing NK cells dysfunction through production of prostaglandin E2 and indoleamine 2,3-dioxygenase in hepatocellular carcinoma (104). These studies revealed that multiple pathways lead to the dysfunction of NK cells and promote the occurrence and development of hepatocellular carcinoma.

Overall, there are several mechanisms of how NK cells protect against the progression of NAFLD (**Figure 1B**) although some studies reported NK cells play the opposite role. As for the role of ILC1s in the liver, a study reported that liver ILC1s is protective against acute liver injury. Intraperitoneally injected with 10% CCl_4_ in corn oil at a dose of 10 uL per gram body weight activates liver ILC1s dependent on DNAM-1 and IL-7R. Activated ILC1s secrete IFN-γ, which is regulated by Adenosine triphosphate (ATP)-purinergic receptor P2X, ligand-gated ion channel, 7(P2RX7) signaling. And then, IFN-γ induces hepatocytes expressing Bcl-Xl, thus protecting mice from CCl_4_-induced acute liver injury (105).These results suggest that liver ILC1s are essential for protecting mice from acute liver injury. Notably, a recent study reported that unlike conventional NK cells which derive from the hematopoietic stem cells in adult bone marrow, liver ILC1s develop from Lin^-^Sca1^+^Mac1^+^ pluripotent hematopoietic hepatocytes which are derived from fetal liver. IFN-γ produced by liver ILC1s themselves promote their *in situ* development by acting on IFN-γR^+^ liver precursor cells, which forms an IFN-γ feedback loop (106). This study revealed that liver ILC1s are different from conventional NK cells in developmental pathway and emphasized the unique immune status of the liver. The role of liver ILC1s in the development of NAFLD still needs to be explored.

### 3.2 ILC2s and ILC3s Are Involved in the Progression of NAFLD

Little is known on the role of ILC2s during hepatic steatosis and NASH. Main findings are related to fibrosis and tissue repair. In both humans and mice with hepatic fibrosis, IL-33 expression is increased. Further, IL-33 is able to cause rodent liver fibrosis. IL-33 activates liver ILC2s and induces ILC2s expansion. The proportion of ILC2s in ILCs is low in normal human liver. However, their number increases when liver fibrosis occurs and is directly related to the severity of the disease (107). Liver ILC2s secretes IL-13 when stimulated by IL-3, IL-25, and TSLP from hepatocytes, HSCs, and Kupffer cells in response to TLR3 stimulation (108). ILC2-derived IL-13 activates HSC through IL-4R and STAT6-dependent signaling and thus mediates hepatic fibrosis (109, 110). These results suggest ILC2s are involved in liver fibrosis. Targeting ILC2s and modulation of IL-33 may be therapeutic strategies for treating liver fibrosis.

In CCl_4_-induced liver fibrosis, the proportion of IL-22^+^ ILC3 and IL-17A^+^ ILC3 subsets markedly increased. A Co-culture experiment with LX-2 cells showed that ILC3s directly promote LX-2 fibrogenesis by IL-17A and IL-22 (111). However, a recent study using *Rorc^gfp/gfp^* mice and *in vitro* primary hepatocytes showed that ILC3s protect from HFD induced steatohepatitis and IL-22 from ILC3s increases lipid metabolism and suppresses apoptosis (112). Besides, IL-22 can regulate lipogenesis related genes and prevent liver steatosis (113). IL-22-Fc treatment restores liver insulin sensitivity, decreased hepatic triglyceride and cholesterol levels, and ameliorated liver steatosis in diet-induce obesity and db/db mice. IL-22-Fc directly functioned on hepatocytes to induce Stat3 activation *in vitro (* 114). This study indicates that ILC3s can protect mice from liver steatosis through producing IL-22. However, whether ILC3s in the liver play a protective or promoting role in the progression of NAFLD is worth further investigation.

## 4 ILCs in Pancreas

Pancreas contains exocrine glands and endocrine glands. Exocrine glands secret pancreatic juice, which has a strong digestion capacity. Endocrine function is performed by specialized cells located in the pancreas. These cells aggregate into clusters and are dispersed in the pancreas, called pancreatic islets. There are four types of hormone-secreting cells in the pancreatic islet, α, β, δ, and F cells. Among them, α cells secrete glucagon and β cells secrete insulin. Insulin plays a wide and complex physiological role in regulation of glucose and lipid metabolism. Type 1 diabetes mellitus has an early onset autoimmune disorder, which leads to failure of insulin secretion. In contrast, Type 2 diabetes mellitus is associated with obesity and insulin resistance. In the early stages of insulin resistance, β-cells compensate by secreting more insulin and increasing β-cell proliferation. As insulin resistance and inflammation prolong, β-cell stress impairs glucose tolerance. Finally, β-cell failure leads to type 2 diabetes. As there are few studies about NK cells and ILC1s in the pancreas in obesity and insulin resistance, we introduce the role of ILC2s and ILC3s in the pancreas.

### 4.1 ILC2s Regulate the Pancreas Function

During obesity, the inflammation in pancreatic islets makes β cells fail to secrete insulin. In lean mice, IL-33 from islet mesenchymal cells activates ILC2s in the pancreas. Activated ILC2s secrete colony-stimulating factor 2 and IL-13, therefore inducing retinoic acid (RA) from macrophages and dendritic cells. Local RA signals promote β cell function and increase insulin secretion. Obesity impairs the IL-33-ILC2 signal and islet function, which can be rescued by IL-33 injection (115). Besides, ILC2s activate tumor immunity to restrict pancreas-specific tumor growth (116). These results suggest that ILC2s can promote insulin secretion. Selective activation of type 2 immunity may be a therapeutic strategy for treating diabetes.

### 4.2 ILC3s Alter the Pancreatic Function

AHR ligands from gut microbiota induce pancreatic ILC3s secreting IL-22 which induces pancreatic endocrine cells expressing β-defensin 14 (mBD14). mBD14 stimulates B cells secreting IL-4, promoting regulatory macrophages and T cells to inhibit autoimmune diabetes (117). This study identified crosstalk between ILCs and endocrine cells in pancreas associated with autoimmune diabetes. Besides, IL-22 administration inhibits islets’ oxidative stress and ER stress, restoring insulin secretion and glucose homeostasis in obese mice (118). This study indicates that ILC3s and IL-22 in the pancreas may play a role in preventing obesity-associated type 2 diabetes. However, this hypothesis still needs to be verified.

## 5 ILCs in Intestine

The gut is an extensive immune system due to exposure to many microorganisms and ingested antigens. The gut microbiota is altered in obesity and its associated metabolic disease, known as dysbiosis (119–121). One major consequence of dysbiosis is defects in the gut barrier, increasing the leakage of bacterial products and contributing to chronic low-grade inflammation and insulin resistance (122–124). As sensor of the microbiota, the intestinal immune system was an essential regulator of obesity-related insulin resistance (125–127). As there are few studies about NK cells and ILC1s in the intestine in obesity, we introduce ILC2s and ILC3s in the intestine and focus on the function and regulation of intestinal ILC3 in metabolism.

### 5.1 ILC2s in the Intestine Induce Obesity

Despite that ILC2s in adipose tissue have the potential to limit obesity, a recent study suggested that ILC2s in the gut induce obesity (81). *Il2rg^−/−^Rag2^−/−^* mice lacking ILCs, T and B cells resist HFD-induced obesity compared with *Rag2^−/−^* mice lacking T and B cells. Adoptive transfer experiment has showed that supplementation of ILC2s from the small intestine could render *Il2rg^-/-^Rag2^-/-^* mice prone to HFD-induced obesity. IL-2 from ILC2s in the small intestine may thus be critical to the induction of obesity and insulin resistance. These results also suggest that the role of ILCs in the regulation of obesity and associated metabolic disease is tissue-specific. The detailed effect of intestinal ILC2s on obesity still needs to be further investigated.

### 5.2 Intestinal ILC3s Produce Cytokines to Regulate Metabolism

ILC3s are abundant in the intestine. Gut ILC3 cells produce the cytokine interleukin-22 (IL-22), which exerts essential roles in eliciting an innate immune response (128), maintaining mucosal barrier integrity (129), and assuring gut homeostasis (130–132). Notably, IL-22 from ILC3s has been demonstrated to improve metabolic disorders. IL-22 from ILCs and CD4^+^ T cells is reduced in obesity under immune challenges. Mice deficient in the IL-22 receptor are more prone to metabolic disorders. Injection of IL-22 can reverse many metabolic symptoms in obese mice. The beneficial effects of IL-22 include preserving gut permeability, reducing endotoxemia and inflammation, regulating lipid metabolism, and improving insulin sensitivity (114). Moreover, IL-22 from ILC3s improves the Polycystic ovary syndrome (PCOS) phenotype. Mice transplanted with stool from PCOS patients display a reduced percentage of IL-22^+^ ILC3s and develop insulin resistance. Administration of glycodeoxycholic acid induces IL-22 secretion from ILC3s through GATA3, which improves the disorder. The mechanisms of IL-22-mediated improvements likely involve promoting adipose tissue browning and inhibiting inflammation (133). Interestingly, exhaustive exercise decreases the proportion of ILC3s and mRNA levels of IL-22 in lamina propria, which destroys intestinal barrier integrity and aggravates intestinal inflammation (134). However, IL-22 can decrease the expression of lipid transporter in the small intestine, which impairs lipid metabolism (135). Consistently, a study using single-cell RNA sequencing has identified a population of DC cells, named CIA-DCs, as the major source of IL-22 binding protein (IL-22BP). Mice lacking IL-22BP demonstrate an increase in functional IL-22. This alteration is associated with the concurrent reduction in the expression of lipid transporters, leading to a decrement in lipid resorption and subsequent change in body fat homeostasis (136). Interestingly, mice feeding carbohydrate diet express higher levels of enzymes and transporters required for carbohydrate digestion and absorption, compared with mice feeding protein diet. γδT cells regulate this process by inhibiting IL-22 production by ILC3s. Treating Organoids with IL-22 reduced the carbohydrate transcriptional program (137). These studies thus indicate that IL-22 from ILC3s regulates nutrition absorption. Overall, the fact that IL-22 from ILC3s regulates metabolism homeostasis highlights the link between metabolism and immunity and provides a new avenue for therapeutic intervention of metabolic diseases.

Although ILC populations and their potentiality of secreting IL-22 are nearly intact in the colon of obese mice, the upstream cytokine IL-23, which activates ILC3 to produce IL-22, is reduced in obese mice after pathogenic bacteria infection (114). Lack of IL-23-IL-22 signaling damaged the intestinal barrier, increasing the concentration of lipopolysaccharide (LPS) in plasma (138). In mice fed HFD, the relative proportion of IL22-producing NKp46^+^ CD4^−^ ILC3s is reduced despite the increase in the total cell numbers of ILC3s in the colon (125). The impairment of IL-23-ILC3-IL22 signaling may partly lead to obesity and insulin resistance.

Another essential cytokine produced by ILC3s is IL-17. IL-17 regulates the migration of intestinal neutrophils, protects the gut barrier, reduces systemic LPS, and improve metabolic syndrome (139). Intestinal IL-17-secreting ILCs can also promote host-microbiota mutualism, preventing liver inflammation and dysfunction of lipid metabolism (140). However, Teijeiro et al. have reported (141) that IL-17A promotes diet-induced obesity and metabolic syndrome. Disruption of IL-17 production or knockdown of IL-17 receptor inhibits diet-induced obesity and metabolic disorders, promoting adipose tissue beigeing, thermogenesis and energy expenditure. Mechanistically, IL-17A induces phosphorylation of the serine 273 site of PPARγ in adipocytes in a Cyclin-dependent Kinase 5 (CDK5)-dependent manner, which subsequently modifies the expression of obesity-associated genes. Interestingly, mothers exposed to HFD render the offspring having more IL-17^+^ ILC3s through microbiota, and increasing the offspring’s susceptibility to intestinal injury. Further, the IL-17 blockade reversed the susceptibility to inflammation (142). Thus, whether IL-17^+^ ILC3s in the intestine are beneficial or adverse to metabolism remains paradoxical, which may depend on specific conditions.

### 5.3 Regulation of Intestinal ILC3s

**Figure 3:**
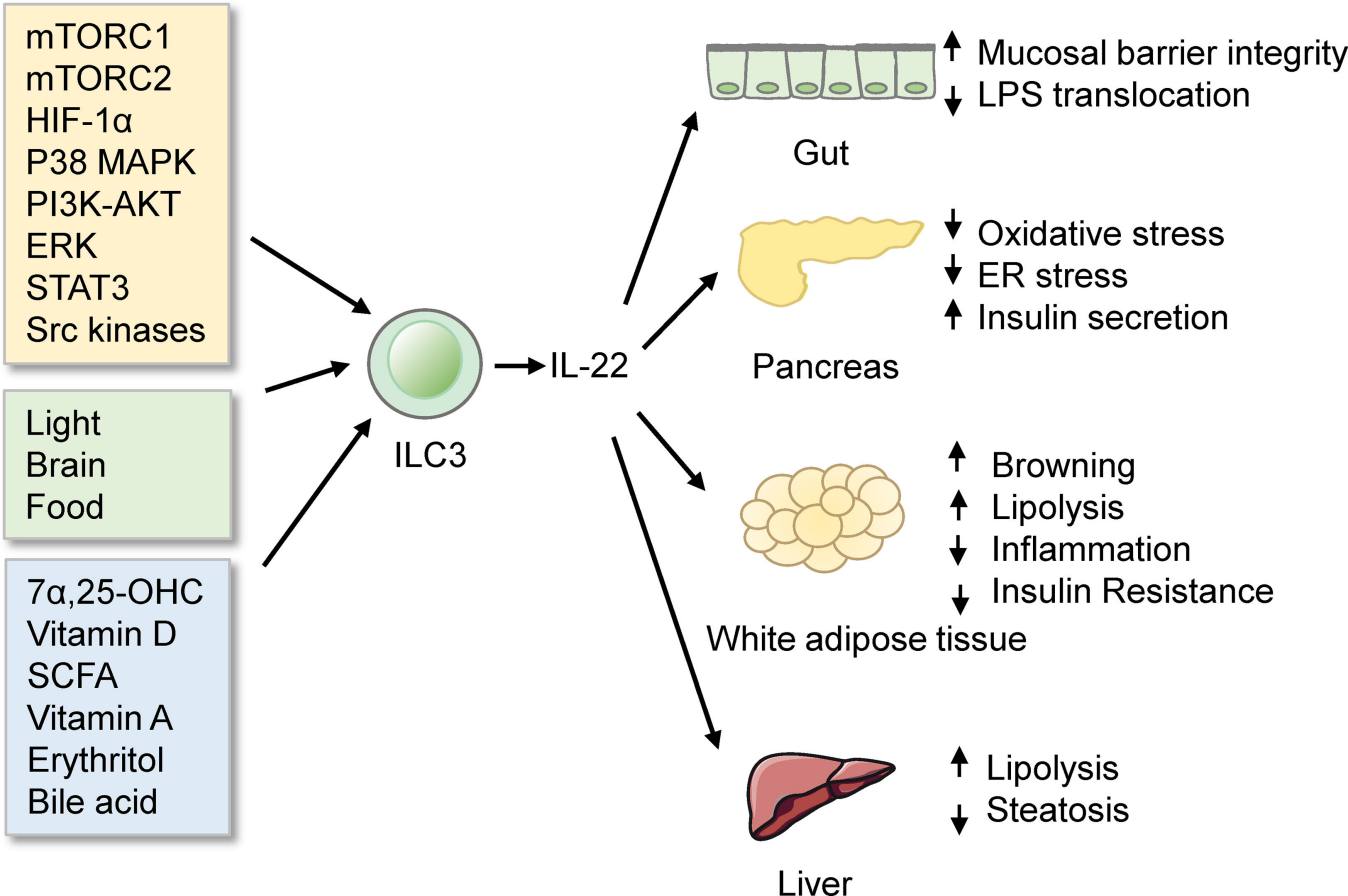
ILC3s in the intestine. ILC3s produce IL-22 to protect mice from obesity and metabolic disorders. ILC3s are regulated by many signals, such as signaling pathways, biological rhythmicity, nutritional signals, and microbiota products.

Multiple signaling pathways regulate ILC3 responses in the intestine (**Figure 3**). Among these modulations, mTOR complex 1 (mTORC1) is critical for the proliferation of ILC3s and production of IL-22 and IL-17A after activation and Citrobacter rodentium infection (143). Moreover, the capacity of ILC3s presenting antigen to T cells is reduced by IL-23, which is also dependent on mTORC1 phosphorylation (144). Recent studies further reveal that both mTORC1 and mTORC2 control ILC3 cell numbers and ILC3-driven inflammation during colitis (145). mTOR signaling influences ILC3s in the intestine, leading to subsequent alteration in metabolic homeostasis. Notably, another study has reported that activation of ILC3s upon low oxygen challenge occurs *via* a HIF-1α-dependent mechanism instead of mTOR-signaling (146). P38 MAPK pathway also regulates the production of GM-CSF by ILC3s after activation of death receptor 3 (DR3) signaling (147, 148). PI3K-AKT or ERK signaling regulates the activation of ILC3s by Lysophosphatidylserine (LysoPS) from apoptotic neutrophils (149). IL-17D acts *via* the CD93 on ILC3s to regulate the production of IL-22 (150), whereas IL-7 activates ILC3s to secret IL-22 through aryl hydrocarbon receptor (AHR) and STAT3 (151). However, a recent study has reported that ILC3-driven tissue repair is IL-22 and STAT3 independent. Instead, this occurs through activation of Src family kinases (152).

Functions of ILC3s in the intestine are influenced by rhythmicity. Environmental light signals regulate intestinal ILC3s functions and further regulate the homeostasis of the intestine and the lipid metabolism in mice (153). This concept is further supported by two distinct reports (154, 155). What’s more, food intake affects the functions of ILC3s. ILC3s express vasoactive intestinal peptide receptor type 2 (VIPR2). Food induced-VIPR2 activation inhibits the secreting of IL-22 of ILC3 and epithelial anti-microbial response, thus enhancing the growth of segmented filamentous bacteria and increasing lipid absorption (156). In contrast, another study finds that VIP markedly increased the production of IL-22 of ILC3s. Lack of VIPR2 impaired the production of IL-22 of ILC3s and made mice more susceptible to DSS-induced gut injury (157). Meanwhile, VIP regulates the recruitment of intestinal ILC3s by increasing the gut-homing receptor CCR9 indirectly (158). Whether the VIP-VIPR2 pathway in ILC3s inhibits or stimulates the production of IL-22 is still controversial.

Nutritional signals also regulate ILC3s. GPR183 and its ligand 7α,25-dihydroxycholesterol (7α,25-OHC) regulate the migration of ILC3s. GPR183-deficient mice have lower IL22^+^ ILC3s in the intestine and increased susceptibility to enteric bacterial infection (159). Vitamin D/vitamin D receptor (VDR) signaling regulates the proliferation and function of ILC3 (160). Since VDR is also a receptor of bile acids (161), bile acids may regulate gut ILC3s through VDR. Besides, colonic ILC3s express the receptor, Ffar2, which can sense microbial metabolites. Ffar2 activation by short-chain fatty acid (SCFA) increases IL-22^+^ ILC3s *via* an AKT and STAT3 axis and modulates gut homeostasis (162–164). What’s more, in Vitamin A-deficient mice, ILC3s are markedly reduced, which makes mice more susceptible to acute bacterial infection (165). Besides, erythritol can increase the number of ILC3s in the small intestine and markedly decrease metabolic disorders such as insulin resistance (166). Whether erythritol influences ILC3s directly or indirectly by short-chain fatty acids still needs further study.

Commensal microbes or their products also regulate the intestinal ILC3s. Symbiotic microbiota represses the production of IL-22 from ILC3s (167), while it indirectly induces the production of GM-CSF and IL-2 from ILC3s by increasing the interleukin-1β (IL-1β) from macrophages. GM-CSF and IL-2 in turn help maintain Treg cell numbers and intestinal homeostasis (168, 169). Besides, gut microbiota regulates ILC3s through bile acid metabolism (161, 170). Glycodeoxycholic acid induces intestinal ILC3s to secrete IL-22, improving insulin resistance (133). Interestingly, ketogenic diets alleviate colitis and reduce the activation of ILC3s (171). Furthermore, single-cell RNA-seq reveals that ILC3s integrate signals from the microbiota to alter phenotypic and functional plasticity (172). The gut microbiome and metabolic syndrome are closely linked (121). Whether gut microbiota influences the metabolism by regulating ILC3s still needs to be investigated.

Overall, ILC3s are involved in the development of obesity and insulin resistance through the production of IL-22 and IL-17. The signals regulating ILC3s may provide novel therapeutic approaches against obesity and metabolic disorders.

## 6 Conclusion and Perspectives

Multiple immune cells in the metabolic organs play diverse roles (**Table 2**). Here, we focus on the functions of ILCs in different metabolic organs in obesity and insulin resistance. In adipose tissue, NK cells and ILC1s trigger macrophage M1 polarization and thus contribute to inflammation, insulin resistance, and even adipose tissue fibrosis.ILC2s produce type 2 cytokines, orchestrate type 2 immunity and maintain metabolic homeostasis. Besides, ILC2s promote white adipose tissue beigeing, increasing energy expenditure and protecting against obesity and insulin resistance. However, the homeostasis of ILC2s is disrupted in obesity. ILC3s are present in human adipose tissue but not mice adipose tissue. The frequency and density of ILC3s increase with the BMI of obese patients. However, further analyses are required to clarify the function and mechanism of ILC3s in human adipose tissue. In the liver, NK cells and ILC1s prevent fibrosis, while ILC2s are profibrotic. These observations suggest a tissue-specific action for ILCs. Whether ILC3s promote or inhibit the progression of NAFLD is worth further investigation. In the pancreas, ILC2s and ILC3s regulate the development of type 2 and type 1 diabetes, respectively. Whether ILC3s in the pancreas are relevant to type 2 diabetes requires further investigation. In the intestine, ILC3s may either improve metabolic disorders through the production of IL-22 or promote metabolic disease by producing IL-17. The intestinal ILC3s are regulated by internal and external signals, which may further influence the homeostasis of the intestine and the metabolism. ILC2s in the gut induce obesity through IL-2.

**Table 2:** Summary of reported roles for adipose tissue immune cells.

Cell types	Produce molecules	Inflammation and Insulin resistance	Beigeing
M1 Macrophages	MCP1, osteopontin, TNFα	promote (173)	inhibit (174)
CD8^+^ T cells	IFN-γ	promote (175, 176)	inhibit (176)
Th1 cells	IFN-γ	promote (177)	N
Th17 cells	IL-17	promote (178)	N
MAIT cells	IL-17	promote (179, 180)	N
B2 cells	IgG2c, TNF, IFN-γ, MCP1, IL-6, IL-8	promote (181–183)	N
Plasmacytoid dendritic cell	IFNα	promote (184)	N
ILC1s	IFN-γ, TNFα	promote (44)	N
NK cells	IFN-γ	promote (41)	N
Mast cells	Serotonin, 15-deoxy-Δ12,14-prostaglandin J2, mast cell protease 6	promote (185)	inhibit (186)
M2 Macrophages	platelet-derived growth factor, matrix metalloproteinases, vascular endothelial growth factor	inhibit (187)	promote
Eosinophils	IL-4, IL-13	inhibit (53)	promote (188)
Th2 cells	IL-4, IL-13	inhibit (177, 189)	N
Treg cells	IL-10	inhibit (190, 191)	promote (190)
γδ T cells	IL-17A and IL-17F	inhibit (192, 193)	promote (192)
iNKT cells	IL-2, IL-4, IL-10	inhibit (194, 195)	promote (195)
B1 cells	IgM and IL-10 proinflammatory IgG	inhibit (181–183)	N
Conventional dendritic cell	IL-10	inhibit (196)	N
ILC2s	IL-5, IL-4, IL-13, Met-Enk	inhibit (54)	promote (60)

N, not determined.

Despite these findings, numerous questions remain unsolved relating to the roles of ILCs on metabolic disease. For example, can ILC3s affect metabolic homeostasis in response to the altered gut microbiota? Do ILC1s suppress adipose tissue beigeing by inducing macrophages’ M1 polarization? What’s the specific role of intestinal ILC2s and ILC3s in developing obesity and insulin resistance? Is there a specific population of ILC3s in the adipose tissue, and if yes, what function do they serve? Addressing these relevant questions will shed new light on the immune regulation of metabolism. Further research investigating the mechanisms of how ILCs influence metabolism may provide novel approaches for intervention of obesity and insulin resistance.

## Author Contributions

HC: Original draft, review, and editing. LS and LF: Review and editing. YY and WZ: Supervision, review, and editing. All authors contributed to the article and approved the submitted version.

## Funding

This research was supported by grants from the National Natural Science Foundation of China (81730020, 81930015, 82070592), the Young Elite Scientist Sponsorship Program by CAST No. YESS20200034 (to YY) and National Institutes of Health Grant R01DK112755, 1R01DK129360 and 1R01DK110273.

## Conflict of Interest

The authors declare that the research was conducted in the absence of any commercial or financial relationships that could be construed as a potential conflict of interest.

## Publisher’s Note

All claims expressed in this article are solely those of the authors and do not necessarily represent those of their affiliated organizations, or those of the publisher, the editors and the reviewers. Any product that may be evaluated in this article, or claim that may be made by its manufacturer, is not guaranteed or endorsed by the publisher.

## References

[1] NCD Risk Factor Collaboration (NCD-RisC). Trends in Adult Body-Mass Index in 200 Countries From 1975 to 2014: A Pooled Analysis of 1698 Population-Based Measurement Studies With 19·2 Million Participants. Lancet (2016) 387:1377–96. doi: 10.1016/S0140-6736(16)30054-X PMC7615134 27115820

[2] NCD Risk Factor Collaboration (NCD-RisC). Worldwide Trends in Body-Mass Index, Underweight, Overweight, and Obesity From 1975 to 2016: A Pooled Analysis of 2416 Population-Based Measurement Studies in 128·9 Million Children, Adolescents, and Adults. Lancet (2017) 390:2627–42. doi: 10.1016/S0140-6736(17)32129-3 PMC5735219 29029897

[3] Global BMI Mortality Collaboration Di Angelantonio E Bhupathiraju S Wormser D Gao P Kaptoge S. Body-Mass Index and All-Cause Mortality: Individual-Participant-Data Meta-Analysis of 239 Prospective Studies in Four Continents. Lancet (2016) 388:776–86. doi: 10.1016/S0140-6736(16)30175-1 PMC4995441 27423262

[4] Pi-Sunyer FX. Comorbidities of Overweight and Obesity: Current Evidence and Research Issues. Med Sci Sports Exerc (1999) 31:S602–608. doi: 10.1097/00005768-199911001-00019 10593535

[5] Lumeng CN Saltiel AR. Inflammatory Links Between Obesity and Metabolic Disease. J Clin Invest (2011) 121:2111–7. doi: 10.1172/JCI57132 PMC3104776 21633179

[6] Saad MJA Santos A Prada PO. Linking Gut Microbiota and Inflammation to Obesity and Insulin Resistance. Physiol (Bethesda) (2016) 31:283–93. doi: 10.1152/physiol.00041.2015 27252163

[7] Nguyen MTA Favelyukis S Nguyen A-K Reichart D Scott PA Jenn A. A Subpopulation of Macrophages Infiltrates Hypertrophic Adipose Tissue and Is Activated by Free Fatty Acids *via* Toll-Like Receptors 2 and 4 and JNK-Dependent Pathways. J Biol Chem (2007) 282:35279–92. doi: 10.1074/jbc.M706762200 17916553

[8] Maqdasy S Lecoutre S Renzi G Frendo-Cumbo S Rizo-Roca D Moritz T. Impaired Phosphocreatine Metabolism in White Adipocytes Promotes Inflammation. Nat Metab (2022) 4: 1–13. doi: 10.1038/s42255-022-00525-9 35165448 PMC8885409

[9] Zick Y. Ser/Thr Phosphorylation of IRS Proteins: A Molecular Basis for Insulin Resistance. Sci STKE (2005) 2005:pe4. doi: 10.1126/stke.2682005pe4 15671481

[10] Hotamisligil GS Shargill NS Spiegelman BM. Adipose Expression of Tumor Necrosis Factor-Alpha: Direct Role in Obesity-Linked Insulin Resistance. Science (1993) 259:87–91. doi: 10.1126/science.7678183 7678183

[11] Weisberg SP McCann D Desai M Rosenbaum M Leibel RL Ferrante AW. Obesity Is Associated With Macrophage Accumulation in Adipose Tissue. J Clin Invest (2003) 112:1796–808. doi: 10.1172/JCI19246 PMC296995 14679176

[12] Mauer J Chaurasia B Goldau J Vogt MC Ruud J Nguyen KD. Signaling by IL-6 Promotes Alternative Activation of Macrophages to Limit Endotoxemia and Obesity-Associated Resistance to Insulin. Nat Immunol (2014) 15:423–30. doi: 10.1038/ni.2865 PMC4161471 24681566

[13] Hotamisligil GS. Inflammation, Metaflammation and Immunometabolic Disorders. Nature (2017) 542:177–85. doi: 10.1038/nature21363 28179656

[14] Brestoff JR Artis D. Regulation of Metabolic Homeostasis in Health and Disease. Cell (2015) 161:146–60. doi: 10.1016/j.cell.2015.02.022 PMC4400287 25815992

[15] McLaughlin T Ackerman SE Shen L Engleman E. Role of Innate and Adaptive Immunity in Obesity-Associated Metabolic Disease. J Clin Invest (2017) 127:5–13. doi: 10.1172/JCI88876 28045397 PMC5199693

[16] Man K Kutyavin VI Chawla A. Tissue Immunometabolism: Development, Physiology, and Pathobiology. Cell Metab (2017) 25:11–26. doi: 10.1016/j.cmet.2016.08.016 27693378 PMC5226870

[17] Lackey DE Olefsky JM. Regulation of Metabolism by the Innate Immune System. Nat Rev Endocrinol (2016) 12:15–28. doi: 10.1038/nrendo.2015.189 26553134

[18] Saltiel AR Olefsky JM. Inflammatory Mechanisms Linking Obesity and Metabolic Disease. J Clin Invest (2017) 127:1–4. doi: 10.1172/JCI92035 28045402 PMC5199709

[19] Lee YS Wollam J Olefsky JM. An Integrated View of Immunometabolism. Cell (2018) 172:22–40. doi: 10.1016/j.cell.2017.12.025 29328913 PMC8451723

[20] Cildir G Akıncılar SC Tergaonkar V. Chronic Adipose Tissue Inflammation: All Immune Cells on the Stage. Trends Mol Med (2013) 19:487–500. doi: 10.1016/j.molmed.2013.05.001 23746697

[21] Gasteiger G Fan X Dikiy S Lee SY Rudensky AY. Tissue Residency of Innate Lymphoid Cells in Lymphoid and Nonlymphoid Organs. Science (2015) 350:981–5. doi: 10.1126/science.aac9593 PMC4720139 26472762

[22] Vivier E Artis D Colonna M Diefenbach A Di Santo JP Eberl G. Innate Lymphoid Cells: 10 Years on. Cell (2018) 174:1054–66. doi: 10.1016/j.cell.2018.07.017 30142344

[23] Spits H Artis D Colonna M Diefenbach A Di Santo JP Eberl G. Innate Lymphoid Cells–a Proposal for Uniform Nomenclature. Nat Rev Immunol (2013) 13:145–9. doi: 10.1038/nri3365 23348417

[24] Eberl G Colonna M Di Santo JP McKenzie ANJ. Innate Lymphoid Cells. Innate Lymphoid Cells: A New Paradigm in Immunology. Science (2015) 348:aaa6566. doi: 10.1126/science.aaa6566 25999512 PMC5658207

[25] Artis D Spits H. The Biology of Innate Lymphoid Cells. Nature (2015) 517:293–301. doi: 10.1038/nature14189 25592534

[26] Klose CSN Artis D. Innate Lymphoid Cells as Regulators of Immunity, Inflammation and Tissue Homeostasis. Nat Immunol (2016) 17:765–74. doi: 10.1038/ni.3489 27328006

[27] Vivier E Tomasello E Baratin M Walzer T Ugolini S. Functions of Natural Killer Cells. Nat Immunol (2008) 9:503–10. doi: 10.1038/ni1582 18425107

[28] Walzer T Bléry M Chaix J Fuseri N Chasson L Robbins SH. Identification, Activation, and Selective *In Vivo* Ablation of Mouse NK Cells *via* Nkp46. Proc Natl Acad Sci U.S.A. (2007) 104:3384–9. doi: 10.1073/pnas.0609692104 PMC1805551 17360655

[29] Arnon TI Markel G Mandelboim O. Tumor and Viral Recognition by Natural Killer Cells Receptors. Semin Cancer Biol (2006) 16:348–58. doi: 10.1016/j.semcancer.2006.07.005 16893656

[30] Weizman O-E Adams NM Schuster IS Krishna C Pritykin Y Lau C. ILC1 Confer Early Host Protection at Initial Sites of Viral Infection. Cell (2017) 171:795–808.e12. doi: 10.1016/j.cell.2017.09.052 29056343 PMC5687850

[31] Moro K Yamada T Tanabe M Takeuchi T Ikawa T Kawamoto H. Innate Production of TH2 Cytokines by Adipose Tissue-Associated C-Kit+Sca-1+ Lymphoid Cells. Nature (2010) 463:540–4. doi: 10.1038/nature08636 20023630

[32] Guia S Narni-Mancinelli E. Helper-Like Innate Lymphoid Cells in Humans and Mice. Trends Immunol (2020) 41:436–52. doi: 10.1016/j.it.2020.03.002 32223931

[33] Takatori H Kanno Y Watford WT Tato CM Weiss G Ivanov II. Lymphoid Tissue Inducer-Like Cells Are an Innate Source of IL-17 and IL-22. J Exp Med (2009) 206:35–41. doi: 10.1084/jem.20072713 19114665 PMC2626689

[34] Hernández PP Mahlakoiv T Yang I Schwierzeck V Nguyen N Guendel F. Interferon-λ and Interleukin 22 Act Synergistically for the Induction of Interferon-Stimulated Genes and Control of Rotavirus Infection. Nat Immunol (2015) 16:698–707. doi: 10.1038/ni.3180 26006013 PMC4589158

[35] Sakers A De Siqueira MK Seale P Villanueva CJ. Adipose-Tissue Plasticity in Health and Disease. Cell (2022) 185:419–46. doi: 10.1016/j.cell.2021.12.016 PMC11152570 35120662

[36] Klein S Gastaldelli A Yki-Järvinen H Scherer PE. Why Does Obesity Cause Diabetes? Cell Metab (2022) 34:11–20. doi: 10.1016/j.cmet.2021.12.012 34986330 PMC8740746

[37] Wu J Boström P Sparks LM Ye L Choi JH Giang A-H. Beige Adipocytes Are a Distinct Type of Thermogenic Fat Cell in Mouse and Human. Cell (2012) 150:366–76. doi: 10.1016/j.cell.2012.05.016 PMC3402601 22796012

[38] Trim WV Lynch L. Immune and Non-Immune Functions of Adipose Tissue Leukocytes. Nat Rev Immunol (2021). doi: 10.1038/s41577-021-00635-7 34741167

[39] Cuff AO Sillito F Dertschnig S Hall A Luong TV Chakraverty R. The Obese Liver Environment Mediates Conversion of NK Cells to a Less Cytotoxic ILC1-Like Phenotype. Front Immunol (2019) 10:2180. doi: 10.3389/fimmu.2019.02180 31572388 PMC6749082

[40] Wensveen FM Jelenčić V Valentić S Šestan M Wensveen TT Theurich S. NK Cells Link Obesity-Induced Adipose Stress to Inflammation and Insulin Resistance. Nat Immunol (2015) 16:376–85. doi: 10.1038/ni.3120 25729921

[41] Lee B-C Kim M-S Pae M Yamamoto Y Eberlé D Shimada T. Adipose Natural Killer Cells Regulate Adipose Tissue Macrophages to Promote Insulin Resistance in Obesity. Cell Metab (2016) 23:685–98. doi: 10.1016/j.cmet.2016.03.002 PMC4833527 27050305

[42] O’Rourke RW Meyer KA Neeley CK Gaston GD Sekhri P Szumowski M. Systemic NK Cell Ablation Attenuates Intra-Abdominal Adipose Tissue Macrophage Infiltration in Murine Obesity. Obes (Silver Spring) (2014) 22:2109–14. doi: 10.1002/oby.20823 PMC4180782 24962029

[43] O’Sullivan TE Rapp M Fan X Weizman O-E Bhardwaj P Adams NM. Adipose-Resident Group 1 Innate Lymphoid Cells Promote Obesity-Associated Insulin Resistance. Immunity (2016) 45:428–41. doi: 10.1016/j.immuni.2016.06.016 PMC5004886 27496734

[44] Boulenouar S Michelet X Duquette D Alvarez D Hogan AE Dold C. Adipose Type One Innate Lymphoid Cells Regulate Macrophage Homeostasis Through Targeted Cytotoxicity. Immunity (2017) 46:273–86. doi: 10.1016/j.immuni.2017.01.008 28228283

[45] Theurich S Tsaousidou E Hanssen R Lempradl AM Mauer J Timper K. IL-6/Stat3-Dependent Induction of a Distinct, Obesity-Associated Nk Cell Subpopulation Deteriorates Energy and Glucose Homeostasis. Cell Metab (2017) 26:171–184.e6. doi: 10.1016/j.cmet.2017.05.018 28683285

[46] Liu F Wang H Feng W Ye X Sun X Jiang C. Type 1 Innate Lymphoid Cells Are Associated With Type 2 Diabetes. Diabetes Metab (2019) 45:341–6. doi: 10.1016/j.diabet.2018.08.005 30189343

[47] Wang H Shen L Sun X Liu F Feng W Jiang C. Adipose Group 1 Innate Lymphoid Cells Promote Adipose Tissue Fibrosis and Diabetes in Obesity. Nat Commun (2019) 10:3254. doi: 10.1038/s41467-019-11270-1 31332184 PMC6646407

[48] Mogilenko DA Caiazzo R L’homme L Pineau L Raverdy V Noulette J. Ifnγ-Producing NK Cells in Adipose Tissue Are Associated With Hyperglycemia and Insulin Resistance in Obese Women. Int J Obes (Lond) (2021) 45:1607–17. doi: 10.1038/s41366-021-00826-1 33934108

[49] Tobin LM Mavinkurve M Carolan E Kinlen D O’Brien EC Little MA. NK Cells in Childhood Obesity Are Activated, Metabolically Stressed, and Functionally Deficient. JCI Insight (2017) 2:e94939. doi: 10.1172/jci.insight.94939 PMC5752310 29263296

[50] Viel S Besson L Charrier E Marçais A Disse E Bienvenu J. Alteration of Natural Killer Cell Phenotype and Function in Obese Individuals. Clin Immunol (2017) 177:12–7. doi: 10.1016/j.clim.2016.01.007 26794911

[51] Michelet X Dyck L Hogan A Loftus RM Duquette D Wei K. Metabolic Reprogramming of Natural Killer Cells in Obesity Limits Antitumor Responses. Nat Immunol (2018) 19:1330–40. doi: 10.1038/s41590-018-0251-7 30420624

[52] Clinthorne JF Beli E Duriancik DM Gardner EM. NK Cell Maturation and Function in C57BL/6 Mice Are Altered by Caloric Restriction. J Immunol (2013) 190:712–22. doi: 10.4049/jimmunol.1201837 PMC4080414 23241894

[53] Pedersen L Idorn M Olofsson GH Lauenborg B Nookaew I Hansen RH. Voluntary Running Suppresses Tumor Growth Through Epinephrine- and IL-6-Dependent NK Cell Mobilization and Redistribution. Cell Metab (2016) 23:554–62. doi: 10.1016/j.cmet.2016.01.011 26895752

[54] Molofsky AB Nussbaum JC Liang H-E Van Dyken SJ Cheng LE Mohapatra A. Innate Lymphoid Type 2 Cells Sustain Visceral Adipose Tissue Eosinophils and Alternatively Activated Macrophages. J Exp Med (2013) 210:535–49. doi: 10.1084/jem.20121964 PMC3600903 23420878

[55] Wu D Molofsky AB Liang H-E Ricardo-Gonzalez RR Jouihan HA Bando JK. Eosinophils Sustain Adipose Alternatively Activated Macrophages Associated With Glucose Homeostasis. Science (2011) 332:243–7. doi: 10.1126/science.1201475 PMC3144160 21436399

[56] Qiu Y Nguyen KD Odegaard JI Cui X Tian X Locksley RM. Eosinophils and Type 2 Cytokine Signaling in Macrophages Orchestrate Development of Functional Beige Fat. Cell (2014) 157:1292–308. doi: 10.1016/j.cell.2014.03.066 PMC4129510 24906148

[57] Hams E Locksley RM McKenzie ANJ Fallon PG. Cutting Edge: IL-25 Elicits Innate Lymphoid Type 2 and Type II NKT Cells That Regulate Obesity in Mice. J Immunol (2013) 191:5349–53. doi: 10.4049/jimmunol.1301176 PMC3847854 24166975

[58] Galle-Treger L Sankaranarayanan I Hurrell BP Howard E Lo R Maazi H. Costimulation of Type-2 Innate Lymphoid Cells by GITR Promotes Effector Function and Ameliorates Type 2 Diabetes. Nat Commun (2019) 10:713. doi: 10.1038/s41467-019-08449-x 30755607 PMC6372786

[59] Newland SA Mohanta S Clément M Taleb S Walker JA Nus M. Type-2 Innate Lymphoid Cells Control the Development of Atherosclerosis in Mice. Nat Commun (2017) 8:15781. doi: 10.1038/ncomms15781 28589929 PMC5467269

[60] Lee M-W Odegaard JI Mukundan L Qiu Y Molofsky AB Nussbaum JC. Activated Type 2 Innate Lymphoid Cells Regulate Beige Fat Biogenesis. Cell (2015) 160:74–87. doi: 10.1016/j.cell.2014.12.011 25543153 PMC4297518

[61] Brestoff JR Kim BS Saenz SA Stine RR Monticelli LA Sonnenberg GF. Group 2 Innate Lymphoid Cells Promote Beiging of White Adipose Tissue and Limit Obesity. Nature (2015) 519:242–6. doi: 10.1038/nature14115 PMC4447235 25533952

[62] Ding X Luo Y Zhang X Zheng H Yang X Yang X. IL-33-Driven ILC2/eosinophil Axis in Fat is Induced by Sympathetic Tone and Suppressed by Obesity. J Endocrinol (2016) 231:35–48. doi: 10.1530/JOE-16-0229 27562191 PMC5003423

[63] Goldberg EL Shchukina I Youm Y-H Ryu S Tsusaka T Young KC. IL-33 Causes Thermogenic Failure In Aging by Expanding Dysfunctional Adipose ILC2. Cell Metab (2021) 33:2277–87.e5. doi: 10.1016/j.cmet.2021.08.004 34473956 PMC9067336

[64] Mahlakõiv T Flamar A-L Johnston LK Moriyama S Putzel GG Bryce PJ. Stromal Cells Maintain Immune Cell Homeostasis in Adipose Tissue *via* Production of Interleukin-33. Sci Immunol (2019) 4:eaax0416. doi: 10.1126/sciimmunol.aax0416 31053655 PMC6766755

[65] Spallanzani RG Zemmour D Xiao T Jayewickreme T Li C Bryce PJ. Distinct Immunocyte-Promoting and Adipocyte-Generating Stromal Components Coordinate Adipose Tissue Immune and Metabolic Tenors. Sci Immunol (2019) 4:eaaw3658. doi: 10.1126/sciimmunol.aaw3658 31053654 PMC6648660

[66] Shan B Shao M Zhang Q An YA Vishvanath L Gupta RK. Cold-Responsive Adipocyte Progenitors Couple Adrenergic Signaling to Immune Cell Activation to Promote Beige Adipocyte Accrual. Genes Dev (2021) 35:1333–8. doi: 10.1101/gad.348762.121 PMC8494206 34531316

[67] Shafiei-Jahani P Hurrell BP Galle-Treger L Helou DG Howard E Painter J. DR3 Stimulation of Adipose Resident ILC2s Ameliorates Type 2 Diabetes Mellitus. Nat Commun (2020) 11:4718. doi: 10.1038/s41467-020-18601-7 32948777 PMC7501856

[68] Fali T Aychek T Ferhat M Jouzeau J-Y Busslinger M Moulin D. Metabolic Regulation by Pparγ is Required for IL-33-Mediated Activation of ILC2s In Lung and Adipose Tissue. Mucosal Immunol (2021) 14:585–93. doi: 10.1038/s41385-020-00351-w 33106586

[69] Karagiannis F Masouleh SK Wunderling K Surendar J Schmitt V Kazakov A. Lipid-Droplet Formation Drives Pathogenic Group 2 Innate Lymphoid Cells in Airway Inflammation. Immunity (2020) 52:620–34.e6. doi: 10.1016/j.immuni.2020.03.003 32268121

[70] Zhao X-Y Zhou L Chen Z Ji Y Peng X Qi L. The Obesity-Induced Adipokine Sst2 Exacerbates Adipose Treg and ILC2 Depletion and Promotes Insulin Resistance. Sci Adv (2020) 6:eaay6191. doi: 10.1126/sciadv.aay6191 32426492 PMC7220368

[71] Okamura T Hashimoto Y Mori J Yamaguchi M Majima S Senmaru T. ILC2s Improve Glucose Metabolism Through the Control of Saturated Fatty Acid Absorption Within Visceral Fat. Front Immunol (2021) 12:669629. doi: 10.3389/fimmu.2021.669629 34305899 PMC8300428

[72] Lin Y Xiao L Cai Q Zhu C Li S Li B. The Chemerin-CMKLR1 Axis Limits Thermogenesis by Controlling a Beige Adipocyte/IL-33/Type 2 Innate Immunity Circuit. Sci Immunol (2021) 6:eabg9698. doi: 10.1126/sciimmunol.abg9698 34330814

[73] Rana BMJ Jou E Barlow JL Rodriguez-Rodriguez N Walker JA Knox C. A Stromal Cell Niche Sustains ILC2-Mediated Type-2 Conditioning in Adipose Tissue. J Exp Med (2019) 216:1999–2009. doi: 10.1084/jem.20190689 31248899 PMC6719433

[74] Chang SK Kohlgruber AC Mizoguchi F Michelet X Wolf BJ Wei K. Stromal Cell Cadherin-11 Regulates Adipose Tissue Inflammation and Diabetes. J Clin Invest (2017) 127:3300–12. doi: 10.1172/JCI86881 PMC5669565 28758901

[75] Cardoso F Klein Wolterink RGJ Godinho-Silva C Domingues RG Ribeiro H da Silva JA. Neuro-Mesenchymal Units Control ILC2 and Obesity *via* a Brain-Adipose Circuit. Nature (2021) 597:410–4. doi: 10.1038/s41586-021-03830-7 PMC7614847 34408322

[76] Moriyama S Brestoff JR Flamar A-L Moeller JB Klose CSN Rankin LC. β2-Adrenergic Receptor-Mediated Negative Regulation of Group 2 Innate Lymphoid Cell Responses. Science (2018) 359:1056–61. doi: 10.1126/science.aan4829 29496881

[77] Molofsky AB Van Gool F Liang H-E Van Dyken SJ Nussbaum JC Lee J. Interleukin-33 and Interferon-γ Counter-Regulate Group 2 Innate Lymphoid Cell Activation During Immune Perturbation. Immunity (2015) 43:161–74. doi: 10.1016/j.immuni.2015.05.019 PMC4512852 26092469

[78] Halim TYF Rana BMJ Walker JA Kerscher B Knolle MD Jolin HE. Tissue-Restricted Adaptive Type 2 Immunity Is Orchestrated by Expression of the Costimulatory Molecule OX40l on Group 2 Innate Lymphoid Cells. Immunity (2018) 48:1195–207.e6. doi: 10.1016/j.immuni.2018.05.003 29907525 PMC6015114

[79] Oldenhove G Boucquey E Taquin A Acolty V Bonetti L Ryffel B. PD-1 Is Involved in the Dysregulation of Type 2 Innate Lymphoid Cells in a Murine Model of Obesity. Cell Rep (2018) 25:2053–60.e4. doi: 10.1016/j.celrep.2018.10.091 30463004

[80] Sabapathy V Stremska ME Mohammad S Corey RL Sharma PR Sharma R. Novel Immunomodulatory Cytokine Regulates Inflammation, Diabetes, and Obesity to Protect From Diabetic Nephropathy. Front Pharmacol (2019) 10:572. doi: 10.3389/fphar.2019.00572 31191312 PMC6540785

[81] Sasaki T Moro K Kubota T Kubota N Kato T Ohno H. Innate Lymphoid Cells in the Induction of Obesity. Cell Rep (2019) 28:202–17.e7. doi: 10.1016/j.celrep.2019.06.016 31269440

[82] Hildreth AD Ma F Wong YY Sun R Pellegrini M O’Sullivan TE. Single-Cell Sequencing of Human White Adipose Tissue Identifies New Cell States in Health and Obesity. Nat Immunol (2021) 22:639–53. doi: 10.1038/s41590-021-00922-4 PMC8102391 33907320

[83] Wang F-S Fan J-G Zhang Z Gao B Wang H-Y. The Global Burden of Liver Disease: The Major Impact of China. Hepatology (2014) 60:2099–108. doi: 10.1002/hep.27406 PMC4867229 25164003

[84] Lazarus JV Ekstedt M Marchesini G Mullen J Novak K Pericàs JM. A Cross-Sectional Study of the Public Health Response to Non-Alcoholic Fatty Liver Disease in Europe. J Hepatol (2020) 72:14–24. doi: 10.1016/j.jhep.2019.08.027 31518646

[85] Younossi ZM Koenig AB Abdelatif D Fazel Y Henry L Wymer M. Global Epidemiology of Nonalcoholic Fatty Liver Disease-Meta-Analytic Assessment of Prevalence, Incidence, and Outcomes. Hepatology (2016) 64:73–84. doi: 10.1002/hep.28431 26707365

[86] Milić S Lulić D Štimac D. Non-Alcoholic Fatty Liver Disease and Obesity: Biochemical, Metabolic and Clinical Presentations. World J Gastroenterol (2014) 20:9330–7. doi: 10.3748/wjg.v20.i28.9330 PMC4110564 25071327

[87] Khan RS Newsome PN. NAFLD in 2017: Novel Insights Into Mechanisms of Disease Progression. Nat Rev Gastroenterol Hepatol (2018) 15:71–2. doi: 10.1038/nrgastro.2017.181 29300050

[88] Luci C Vieira E Perchet T Gual P Golub R. Natural Killer Cells and Type 1 Innate Lymphoid Cells Are New Actors in Non-Alcoholic Fatty Liver Disease. Front Immunol (2019) 10:1192. doi: 10.3389/fimmu.2019.01192 31191550 PMC6546848

[89] Tian Z Chen Y Gao B. Natural Killer Cells in Liver Disease. Hepatology (2013) 57:1654–62. doi: 10.1002/hep.26115 PMC3573257 23111952

[90] Wu J Wu D Zhang L Lin C Liao J Xie R. NK Cells Induce Hepatic ER Stress to Promote Insulin Resistance in Obesity Through Osteopontin Production. J Leukoc Biol (2020) 107:589–96. doi: 10.1002/JLB.3MA1119-173R 31829469

[91] Tosello-Trampont A-C Krueger P Narayanan S Landes SG Leitinger N Hahn YS. NKp46+ Natural Killer Cells Attenuate Metabolism-Induced Hepatic Fibrosis by Regulating Macrophage Activation in Mice. Hepatology (2016) 63:799–812. doi: 10.1002/hep.28389 26662852 PMC4764418

[92] Idrissova L Malhi H Werneburg NW LeBrasseur NK Bronk SF Fingas C. TRAIL Receptor Deletion in Mice Suppresses the Inflammation of Nutrient Excess. J Hepatol (2015) 62:1156–63. doi: 10.1016/j.jhep.2014.11.033 PMC4404200 25445398

[93] Stiglund N Strand K Cornillet M Stål P Thorell A Zimmer CL. Retained NK Cell Phenotype and Functionality in Non-Alcoholic Fatty Liver Disease. Front Immunol (2019) 10:1255. doi: 10.3389/fimmu.2019.01255 31214196 PMC6558016

[94] Melhem A Muhanna N Bishara A Alvarez CE Ilan Y Bishara T. Anti-Fibrotic Activity of NK Cells in Experimental Liver Injury Through Killing of Activated HSC. J Hepatol (2006) 45:60–71. doi: 10.1016/j.jhep.2005.12.025 16515819

[95] Mederacke I Hsu CC Troeger JS Huebener P Mu X Dapito DH. Fate Tracing Reveals Hepatic Stellate Cells as Dominant Contributors to Liver Fibrosis Independent of Its Aetiology. Nat Commun (2013) 4:2823. doi: 10.1038/ncomms3823 24264436 PMC4059406

[96] Krizhanovsky V Yon M Dickins RA Hearn S Simon J Miething C. Senescence of Activated Stellate Cells Limits Liver Fibrosis. Cell (2008) 134:657–67. doi: 10.1016/j.cell.2008.06.049 PMC3073300 18724938

[97] Radaeva S Sun R Jaruga B Nguyen VT Tian Z Gao B. Natural Killer Cells Ameliorate Liver Fibrosis by Killing Activated Stellate Cells in NKG2D-Dependent and Tumor Necrosis Factor-Related Apoptosis-Inducing Ligand-Dependent Manners. Gastroenterology (2006) 130:435–52. doi: 10.1053/j.gastro.2005.10.055 16472598

[98] Gur C Doron S Kfir-Erenfeld S Horwitz E Abu-Tair L Safadi R. NKp46-Mediated Killing of Human and Mouse Hepatic Stellate Cells Attenuates Liver Fibrosis. Gut (2012) 61:885–93. doi: 10.1136/gutjnl-2011-301400 22198715

[99] Li T Yang Y Song H Li H Cui A Liu Y. Activated NK Cells Kill Hepatic Stellate Cells *via* P38/PI3K Signaling in a TRAIL-Involved Degranulation Manner. J Leukoc Biol (2019) 105:695–704. doi: 10.1002/JLB.2A0118-031RR 30748035

[100] Liu P Chen L Zhang H. Natural Killer Cells in Liver Disease and Hepatocellular Carcinoma and the NK Cell-Based Immunotherapy. J Immunol Res (2018) 2018:1206737. doi: 10.1155/2018/1206737 30255103 PMC6142725

[101] Fathy A Eldin MM Metwally L Eida M Abdel-Rehim M. Diminished Absolute Counts of CD56dim and CD56bright Natural Killer Cells in Peripheral Blood From Egyptian Patients With Hepatocellular Carcinoma. Egypt J Immunol (2009) 16:17–25. 22059350

[102] Wu Y Kuang D-M Pan W-D Wan Y-L Lao X-M Wang D. Monocyte/macrophage-Elicited Natural Killer Cell Dysfunction in Hepatocellular Carcinoma Is Mediated by CD48/2B4 Interactions. Hepatology (2013) 57:1107–16. doi: 10.1002/hep.26192 23225218

[103] Hoechst B Voigtlaender T Ormandy L Gamrekelashvili J Zhao F Wedemeyer H. Myeloid Derived Suppressor Cells Inhibit Natural Killer Cells in Patients With Hepatocellular Carcinoma *via* the NKp30 Receptor. Hepatology (2009) 50:799–807. doi: 10.1002/hep.23054 19551844 PMC6357774

[104] Li T Yang Y Hua X Wang G Liu W Jia C. Hepatocellular Carcinoma-Associated Fibroblasts Trigger NK Cell Dysfunction *via* PGE2 and IDO. Cancer Lett (2012) 318:154–61. doi: 10.1016/j.canlet.2011.12.020 22182446

[105] Nabekura T Riggan L Hildreth AD O’Sullivan TE Shibuya A. Type 1 Innate Lymphoid Cells Protect Mice From Acute Liver Injury *via* Interferon-γ Secretion for Upregulating Bcl-xL Expression in Hepatocytes. Immunity (2020) 52:96–108.e9. doi: 10.1016/j.immuni.2019.11.004 31810881 PMC8108607

[106] Bai L Vienne M Tang L Kerdiles Y Etiennot M Escalière B. Liver Type 1 Innate Lymphoid Cells Develop Locally Via an Interferon-γ-Dependent Loop. Science (2021) 371:eaba4177. doi: 10.1126/science.aba4177 33766856

[107] Gonzalez-Polo V Pucci-Molineris M Cervera V Gambaro S Yantorno SE Descalzi V. Group 2 Innate Lymphoid Cells Exhibit Progressively Higher Levels of Activation During Worsening of Liver Fibrosis. Ann Hepatol (2019) 18:366–72. doi: 10.1016/j.aohep.2018.12.001 31053540

[108] Forkel M Berglin L Kekäläinen E Carlsson A Svedin E Michaëlsson J. Composition and Functionality of the Intrahepatic Innate Lymphoid Cell-Compartment in Human Nonfibrotic and Fibrotic Livers. Eur J Immunol (2017) 47:1280–94. doi: 10.1002/eji.201646890 28613415

[109] McHedlidze T Waldner M Zopf S Walker J Rankin AL Schuchmann M. Interleukin-33-Dependent Innate Lymphoid Cells Mediate Hepatic Fibrosis. Immunity (2013) 39:357–71. doi: 10.1016/j.immuni.2013.07.018 PMC4172965 23954132

[110] Vannella KM Ramalingam TR Borthwick LA Barron L Hart KM Thompson RW. Combinatorial Targeting of TSLP, IL-25, and IL-33 in Type 2 Cytokine-Driven Inflammation and Fibrosis. Sci Transl Med (2016) 8:337ra65. doi: 10.1126/scitranslmed.aaf1938 27147589

[111] Wang S Li J Wu S Cheng L Shen Y Ma W. Type 3 Innate Lymphoid Cell: A New Player in Liver Fibrosis Progression. Clin Sci (2018) 132:2565–82. doi: 10.1042/CS20180482 30459204

[112] Hamaguchi M Okamura T Fukuda T Nishida K Yoshimura Y Hashimoto Y. Group 3 Innate Lymphoid Cells Protect Steatohepatitis From High-Fat Diet Induced Toxicity. Front Immunol (2021) 12:648754. doi: 10.3389/fimmu.2021.648754 33790913 PMC8005651

[113] Yang L Zhang Y Wang L Fan F Zhu L Li Z. Amelioration of High Fat Diet Induced Liver Lipogenesis and Hepatic Steatosis by Interleukin-22. J Hepatol (2010) 53:339–47. doi: 10.1016/j.jhep.2010.03.004 20452699

[114] Wang X Ota N Manzanillo P Kates L Zavala-Solorio J Eidenschenk C. Interleukin-22 Alleviates Metabolic Disorders and Restores Mucosal Immunity in Diabetes. Nature (2014) 514:237–41. doi: 10.1038/nature13564 25119041

[115] Dalmas E Lehmann FM Dror E Wueest S Thienel C Borsigova M. Interleukin-33-Activated Islet-Resident Innate Lymphoid Cells Promote Insulin Secretion Through Myeloid Cell Retinoic Acid Production. Immunity (2017) 47:928–42.e7. doi: 10.1016/j.immuni.2017.10.015 29166590

[116] Moral J Lueng J Rojas L Ruan J Zhao J Ssethna Z. ILC2s Amplify PD-1 Blockade by Activating Tissue-Specific Cancer Immunity. Nature (2020) 579:130–5. doi: 10.1038/s41586-020-2015-4 PMC7060130 32076273

[117] Miani M Le Naour J Waeckel-Enée E Verma SC Straube M Emond P. Gut Microbiota-Stimulated Innate Lymphoid Cells Support β-Defensin 14 Expression in Pancreatic Endocrine Cells, Preventing Autoimmune Diabetes. Cell Metab (2018) 28:557–72.e6. doi: 10.1016/j.cmet.2018.06.012 30017352

[118] Hasnain SZ Borg DJ Harcourt BE Tong H Sheng YH Ng CP. Glycemic Control in Diabetes Is Restored by Therapeutic Manipulation of Cytokines That Regulate Beta Cell Stress. Nat Med (2014) 20:1417–26. doi: 10.1038/nm.3705 25362253

[119] Nicholson JK Holmes E Kinross J Burcelin R Gibson G Jia W. Host-Gut Microbiota Metabolic Interactions. Science (2012) 336:1262–7. doi: 10.1126/science.1223813 22674330

[120] Holmes E Li JV Marchesi JR Nicholson JK. Gut Microbiota Composition and Activity in Relation to Host Metabolic Phenotype and Disease Risk. Cell Metab (2012) 16:559–64. doi: 10.1016/j.cmet.2012.10.007 23140640

[121] Dabke K Hendrick G Devkota S. The Gut Microbiome and Metabolic Syndrome. J Clin Invest (2019) 129:4050–7. doi: 10.1172/JCI129194 PMC6763239 31573550

[122] Cani PD Amar J Iglesias MA Poggi M Knauf C Bastelica D. Metabolic Endotoxemia Initiates Obesity and Insulin Resistance. Diabetes (2007) 56:1761–72. doi: 10.2337/db06-1491 17456850

[123] Cani PD Bibiloni R Knauf C Waget A Neyrinck AM Delzenne NM. Changes in Gut Microbiota Control Metabolic Endotoxemia-Induced Inflammation in High-Fat Diet-Induced Obesity and Diabetes in Mice. Diabetes (2008) 57:1470–81. doi: 10.2337/db07-1403 18305141

[124] Amar J Chabo C Waget A Klopp P Vachoux C Bermúdez-Humarán LG. Intestinal Mucosal Adherence and Translocation of Commensal Bacteria at the Early Onset of Type 2 Diabetes: Molecular Mechanisms and Probiotic Treatment. EMBO Mol Med (2011) 3:559–72. doi: 10.1002/emmm.201100159 PMC3265717 21735552

[125] Luck H Tsai S Chung J Clemente-Casares X Ghazarian M Revelo XS. Regulation of Obesity-Related Insulin Resistance With Gut Anti-Inflammatory Agents. Cell Metab (2015) 21:527–42. doi: 10.1016/j.cmet.2015.03.001 25863246

[126] Monteiro-Sepulveda M Touch S Mendes-Sá C André S Poitou C Allatif O. Jejunal T Cell Inflammation in Human Obesity Correlates With Decreased Enterocyte Insulin Signaling. Cell Metab (2015) 22:113–24. doi: 10.1016/j.cmet.2015.05.020 26094890

[127] Garidou L Pomié C Klopp P Waget A Charpentier J Aloulou M. The Gut Microbiota Regulates Intestinal CD4 T Cells Expressing Rorγt and Controls Metabolic Disease. Cell Metab (2015) 22:100–12. doi: 10.1016/j.cmet.2015.06.001 26154056

[128] Muñoz M Eidenschenk C Ota N Wong K Lohmann U Kühl AA. Interleukin-22 Induces Interleukin-18 Expression From Epithelial Cells During Intestinal Infection. Immunity (2015) 42:321–31. doi: 10.1016/j.immuni.2015.01.011 25680273

[129] Sonnenberg GF Fouser LA Artis D. Border Patrol: Regulation of Immunity, Inflammation and Tissue Homeostasis at Barrier Surfaces by IL-22. Nat Immunol (2011) 12:383–90. doi: 10.1038/ni.2025 21502992

[130] Gronke K Hernández PP Zimmermann J Klose CSN Kofoed-Branzk M Guendel F. Interleukin-22 Protects Intestinal Stem Cells Against Genotoxic Stress. Nature (2019) 566:249–53. doi: 10.1038/s41586-019-0899-7 PMC6420091 30700914

[131] Lindemans CA Calafiore M Mertelsmann AM O’Connor MH Dudakov JA Jenq RR. Interleukin-22 Promotes Intestinal-Stem-Cell-Mediated Epithelial Regeneration. Nature (2015) 528:560–4. doi: 10.1038/nature16460 PMC4720437 26649819

[132] Sonnenberg GF Monticelli LA Alenghat T Fung TC Hutnick NA Kunisawa J. Innate Lymphoid Cells Promote Anatomical Containment of Lymphoid-Resident Commensal Bacteria. Science (2012) 336:1321–5. doi: 10.1126/science.1222551 PMC3659421 22674331

[133] Qi X Yun C Sun L Xia J Wu Q Wang Y. Gut Microbiota–Bile Acid–Interleukin-22 Axis Orchestrates Polycystic Ovary Syndrome. Nat Med (2019) 25:1225–33. doi: 10.1038/s41591-019-0509-0 PMC7376369 31332392

[134] Hou P Zhou X Yu L Yao Y Zhang Y Huang Y. Exhaustive Exercise Induces Gastrointestinal Syndrome Through Reduced Ilc3 and Il-22 in Mouse Model. Med Sci Sports Exerc (2020) 52:1710–8. doi: 10.1249/MSS.0000000000002298 32079925

[135] Mao K Baptista AP Tamoutounour S Zhuang L Bouladoux N Martins AJ. Innate and Adaptive Lymphocytes Sequentially Shape the Gut Microbiota and Lipid Metabolism. Nature (2018) 554:255–9. doi: 10.1038/nature25437 29364878

[136] Guendel F Kofoed-Branzk M Gronke K Tizian C Witkowski M Cheng H-W. Group 3 Innate Lymphoid Cells Program a Distinct Subset of IL-22bp-Producing Dendritic Cells Demarcating Solitary Intestinal Lymphoid Tissues. Immunity (2020) 53:1015–32.e8. doi: 10.1016/j.immuni.2020.10.012 33207209

[137] Sullivan ZA Khoury-Hanold W Lim J Smillie C Biton M Reis BS. γδ T Cells Regulate the Intestinal Response to Nutrient Sensing. Science (2021) 371:eaba8310. doi: 10.1126/science.aba8310 33737460 PMC11617329

[138] Fatkhullina AR Peshkova IO Dzutsev A Aghayev T McCulloch JA Thovarai V. An Interleukin-23-Interleukin-22 Axis Regulates Intestinal Microbial Homeostasis to Protect From Diet-Induced Atherosclerosis. Immunity (2018) 49:943–57.e9. doi: 10.1016/j.immuni.2018.09.011 30389414 PMC6257980

[139] Pérez MM Martins LMS Dias MS Pereira CA Leite JA Gonçalves ECS. Interleukin-17/Interleukin-17 Receptor Axis Elicits Intestinal Neutrophil Migration, Restrains Gut Dysbiosis and Lipopolysaccharide Translocation in High-Fat Diet-Induced Metabolic Syndrome Model. Immunology (2019) 156:339–55. doi: 10.1111/imm.13028 PMC6418416 30472727

[140] Martínez-López M Iborra S Conde-Garrosa R Mastrangelo A Danne C Mann ER. Microbiota Sensing by Mincle-Syk Axis in Dendritic Cells Regulates Interleukin-17 and -22 Production and Promotes Intestinal Barrier Integrity. Immunity (2019) 50:446–61.e9. doi: 10.1016/j.immuni.2018.12.020 30709742 PMC6382412

[141] Teijeiro A Garrido A Ferre A Perna C Djouder N. Inhibition of the IL-17A Axis in Adipocytes Suppresses Diet-Induced Obesity and Metabolic Disorders in Mice. Nat Metab (2021) 3:496–512. doi: 10.1038/s42255-021-00371-1 33859430

[142] Babu ST Niu X Raetz M Savani RC Hooper LV Mirpuri J. Maternal High-Fat Diet Results in Microbiota-Dependent Expansion of ILC3s in Mice Offspring. JCI Insight (2018) 3:e99223. doi: 10.1172/jci.insight.99223 PMC6237468 30282818

[143] Di Luccia B Gilfillan S Cella M Colonna M Huang SC-C. ILC3s Integrate Glycolysis and Mitochondrial Production of Reactive Oxygen Species to Fulfill Activation Demands. J Exp Med (2019) 216:2231–41. doi: 10.1084/jem.20180549 PMC6781001 31296736

[144] Lehmann FM von Burg N Ivanek R Teufel C Horvath E Peter A. Microbiota-Induced Tissue Signals Regulate ILC3-Mediated Antigen Presentation. Nat Commun (2020) 11:1794. doi: 10.1038/s41467-020-15612-2 32286285 PMC7156681

[145] Teufel C Horvath E Peter A Ercan C Piscuoglio S Hall MN. mTOR Signaling Mediates ILC3-Driven Immunopathology. Mucosal Immunol (2021) 14:1323–34. doi: 10.1038/s41385-021-00432-4 PMC8528695 34341503

[146] Fachi JL Pral LP Dos Santos JAC Codo AC de Oliveira S Felipe JS. Hypoxia Enhances ILC3 Responses Through HIF-1α-Dependent Mechanism. Mucosal Immunol (2021) 14:828–41. doi: 10.1038/s41385-020-00371-6 PMC8221997 33446906

[147] Li J Shi W Sun H Ji Y Chen Y Guo X. Activation of DR3 Signaling Causes Loss of ILC3s and Exacerbates Intestinal Inflammation. Nat Commun (2019) 10:3371. doi: 10.1038/s41467-019-11304-8 31358760 PMC6662828

[148] Chang Y Kim JW Yang S Chung DH Ko JS Moon JS. Increased GM-CSF-Producing NCR- ILC3s and Neutrophils in the Intestinal Mucosa Exacerbate Inflammatory Bowel Disease. Clin Transl Immunol (2021) 10:e1311. doi: 10.1002/cti2.1311 PMC8264747 34262760

[149] Wang X Cai J Lin B Ma M Tao Y Zhou Y. GPR34-Mediated Sensing of Lysophosphatidylserine Released by Apoptotic Neutrophils Activates Type 3 Innate Lymphoid Cells to Mediate Tissue Repair. Immunity (2021) 54:1123–1136.e8. doi: 10.1016/j.immuni.2021.05.007 34107271

[150] Huang J Lee H-Y Zhao X Han J Su Y Sun Q. Interleukin-17D Regulates Group 3 Innate Lymphoid Cell Function Through Its Receptor CD93. Immunity (2021) 54:673–686.e4. doi: 10.1016/j.immuni.2021.03.018 33852831

[151] Zhu Y Shi T Lu X Xu Z Qu J Zhang Z. Fungal-Induced Glycolysis in Macrophages Promotes Colon Cancer by Enhancing Innate Lymphoid Cell Secretion of IL-22. EMBO J (2021) 40:e105320. doi: 10.15252/embj.2020105320 33591591 PMC8167358

[152] Romera-Hernández M Aparicio-Domingo P Papazian N Karrich JJ Cornelissen F Hoogenboezem RM. Yap1-Driven Intestinal Repair Is Controlled by Group 3 Innate Lymphoid Cells. Cell Rep (2020) 30:37–45.e3. doi: 10.1016/j.celrep.2019.11.115 31914395

[153] Godinho-Silva C Domingues RG Rendas M Raposo B Ribeiro H da Silva JA. Light-Entrained and Brain-Tuned Circadian Circuits Regulate ILC3s and Gut Homeostasis. Nature (2019) 574:254–8. doi: 10.1038/s41586-019-1579-3 PMC6788927 31534216

[154] Teng F Goc J Zhou L Chu C Shah MA Eberl G. A Circadian Clock Is Essential for Homeostasis of Group 3 Innate Lymphoid Cells in the Gut. Sci Immunol (2019) 4:eaax1215. doi: 10.1126/sciimmunol.aax1215 31586011 PMC7008004

[155] Wang Q Robinette ML Billon C Collins PL Bando JK Fachi JL. Circadian Rhythm-Dependent and Circadian Rhythm-Independent Impacts of the Molecular Clock on Type 3 Innate Lymphoid Cells. Sci Immunol (2019) 4:eaay7501. doi: 10.1126/sciimmunol.aay7501 31586012 PMC6911370

[156] Talbot J Hahn P Kroehling L Nguyen H Li D Littman DR. Feeding-Dependent VIP Neuron-ILC3 Circuit Regulates the Intestinal Barrier. Nature (2020) 579:575–80. doi: 10.1038/s41586-020-2039-9 PMC7135938 32050257

[157] Seillet C Luong K Tellier J Jacquelot N Shen RD Hickey P. The Neuropeptide VIP Confers Anticipatory Mucosal Immunity by Regulating ILC3 Activity. Nat Immunol (2019) 21:168–77. doi: 10.1038/s41590-019-0567-y 31873294

[158] Yu HB Yang H Allaire JM Ma C Graef FA Mortha A. Vasoactive Intestinal Peptide Promotes Host Defense Against Enteric Pathogens by Modulating the Recruitment of Group 3 Innate Lymphoid Cells. Proc Natl Acad Sci USA (2021) 118:e2106634118. doi: 10.1073/pnas.2106634118 34625492 PMC8521691

[159] Chu C Moriyama S Li Z Zhou L Flamar A-L Klose CSN. Anti-Microbial Functions of Group 3 Innate Lymphoid Cells in Gut-Associated Lymphoid Tissues Are Regulated by G-Protein-Coupled Receptor 183. Cell Rep (2018) 23:3750–8. doi: 10.1016/j.celrep.2018.05.099 PMC6209103 29949760

[160] He L Zhou M Li YC. Vitamin D/Vitamin D Receptor Signaling Is Required for Normal Development and Function of Group 3 Innate Lymphoid Cells in the Gut. iScience (2019) 17:119–31. doi: 10.1016/j.isci.2019.06.026 PMC6610723 31272068

[161] Song X Sun X Oh SF Wu M Zhang Y Zheng W. Microbial Bile Acid Metabolites Modulate Gut Rorγ+ Regulatory T Cell Homeostasis. Nature (2020) 577:410–5. doi: 10.1038/s41586-019-1865-0 PMC7274525 31875848

[162] Chun E Lavoie S Fonseca-Pereira D Bae S Michaud M Hoveyda HR. Metabolite-Sensing Receptor Ffar2 Regulates Colonic Group 3 Innate Lymphoid Cells and Gut Immunity. Immunity (2019) 51:871–84.e6. doi: 10.1016/j.immuni.2019.09.014 PMC6901086 31628054

[163] Sepahi A Liu Q Friesen L Kim CH. Dietary Fiber Metabolites Regulate Innate Lymphoid Cell Responses. Mucosal Immunol (2021) 14:317–30. doi: 10.1038/s41385-020-0312-8 PMC7736174 32541842

[164] Fachi JL Sécca C Rodrigues PB de Mato FCP Di Luccia B Felipe J de S. Acetate Coordinates Neutrophil and ILC3 Responses Against C. Difficile Through FFAR2. J Exp Med (2020) 217:jem.20190489. doi: 10.1084/jem.20190489 PMC7062529 31876919

[165] Spencer SP Wilhelm C Yang Q Hall JA Bouladoux N Boyd A. Adaptation of Innate Lymphoid Cells to a Micronutrient Deficiency Promotes Type 2 Barrier Immunity. Science (2014) 343:432–7. doi: 10.1126/science.1247606 PMC4313730 24458645

[166] Kawano R Okamura T Hashimoto Y Majima S Senmaru T Ushigome E. Erythritol Ameliorates Small Intestinal Inflammation Induced by High-Fat Diets and Improves Glucose Tolerance. Int J Mol Sci (2021) 22:5558. doi: 10.3390/ijms22115558 34074061 PMC8197374

[167] Sawa S Lochner M Satoh-Takayama N Dulauroy S Bérard M Kleinschek M. Rorγt+ Innate Lymphoid Cells Regulate Intestinal Homeostasis by Integrating Negative Signals From the Symbiotic Microbiota. Nat Immunol (2011) 12:320–6. doi: 10.1038/ni.2002 21336274

[168] Mortha A Chudnovskiy A Hashimoto D Bogunovic M Spencer SP Belkaid Y. Microbiota-Dependent Crosstalk Between Macrophages and Ilc3 Promotes Intestinal Homeostasis. Science (2014) 343:1249288. doi: 10.1126/science.1249288 24625929 PMC4291125

[169] Zhou L Chu C Teng F Bessman NJ Goc J Santosa EK. Innate Lymphoid Cells Support Regulatory T Cells in the Intestine Through Interleukin-2. Nature (2019) 568:405–9. doi: 10.1038/s41586-019-1082-x PMC6481643 30944470

[170] Hang S Paik D Yao L Kim E Trinath J Lu J. Bile Acid Metabolites Control TH17 and Treg Cell Differentiation. Nature (2019) 576:143–8. doi: 10.1038/s41586-019-1785-z PMC6949019 31776512

[171] Kong C Yan X Liu Y Huang L Zhu Y He J. Ketogenic Diet Alleviates Colitis by Reduction of Colonic Group 3 Innate Lymphoid Cells Through Altering Gut Microbiome. Signal Transduct Target Ther (2021) 6:154. doi: 10.1038/s41392-021-00549-9 33888680 PMC8062677

[172] Gury-BenAri M Thaiss CA Serafini N Winter DR Giladi A Lara-Astiaso D. The Spectrum and Regulatory Landscape of Intestinal Innate Lymphoid Cells Are Shaped by the Microbiome. Cell (2016) 166:1231–46.e13. doi: 10.1016/j.cell.2016.07.043 27545347

[173] Xu X Grijalva A Skowronski A van Eijk M Serlie MJ Ferrante AW. Obesity Activates a Program of Lysosomal-Dependent Lipid Metabolism in Adipose Tissue Macrophages Independently of Classic Activation. Cell Metab (2013) 18:816–30. doi: 10.1016/j.cmet.2013.11.001 PMC3939841 24315368

[174] Wolf Y Boura-Halfon S Cortese N Haimon Z Sar Shalom H Kuperman Y. Brown-Adipose-Tissue Macrophages Control Tissue Innervation and Homeostatic Energy Expenditure. Nat Immunol (2017) 18:665–74. doi: 10.1038/ni.3746 PMC5438596 28459435

[175] Nishimura S Manabe I Nagasaki M Eto K Yamashita H Ohsugi M. CD8+ Effector T Cells Contribute to Macrophage Recruitment and Adipose Tissue Inflammation in Obesity. Nat Med (2009) 15:914–20. doi: 10.1038/nm.1964 19633658

[176] Moysidou M Karaliota S Kodela E Salagianni M Koutmani Y Katsouda A. CD8+ T Cells in Beige Adipogenesis and Energy Homeostasis. JCI Insight (2018) 3:e95456. doi: 10.1172/jci.insight.95456 PMC5922290 29515042

[177] McLaughlin T Liu L-F Lamendola C Shen L Morton J Rivas H. T-Cell Profile in Adipose Tissue Is Associated With Insulin Resistance and Systemic Inflammation in Humans. Arterioscler Thromb Vasc Biol (2014) 34:2637–43. doi: 10.1161/ATVBAHA.114.304636 PMC4445971 25341798

[178] Identification of Adipose Tissue Dendritic Cells Correlated With Obesity-Associated Insulin-Resistance and Inducing Th17 Responses in Mice and Patients. In: Diabetes American Diabetes Association. Available at: https://diabetesjournals.org/diabetes/article/61/9/2238/15049/Identification-of-Adipose-Tissue-Dendritic-Cells (Accessed March 8, 2022). 10.2337/db11-1274 PMC3425417 22596049

[179] Toubal A Kiaf B Beaudoin L Cagninacci L Rhimi M Fruchet B. Mucosal-Associated Invariant T Cells Promote Inflammation and Intestinal Dysbiosis Leading to Metabolic Dysfunction During Obesity. Nat Commun (2020) 11:3755. doi: 10.1038/s41467-020-17307-0 32709874 PMC7381641

[180] Magalhaes I Pingris K Poitou C Bessoles S Venteclef N Kiaf B. Mucosal-Associated Invariant T Cell Alterations in Obese and Type 2 Diabetic Patients. J Clin Invest (2015) 125:1752–62. doi: 10.1172/JCI78941 PMC4396481 25751065

[181] Frasca D Blomberg BB. Adipose Tissue Inflammation Induces B Cell Inflammation and Decreases B Cell Function in Aging. Front Immunol (2017) 8:1003. doi: 10.3389/fimmu.2017.01003 28894445 PMC5581329

[182] Srikakulapu P McNamara CA. B Lymphocytes and Adipose Tissue Inflammation. Arterioscler Thromb Vasc Biol (2020) 40:1110–22. doi: 10.1161/ATVBAHA.119.312467 PMC7398177 32131612

[183] Winer DA Winer S Shen L Wadia PP Yantha J Paltser G. B Cells Promote Insulin Resistance Through Modulation of T Cells and Production of Pathogenic IgG Antibodies. Nat Med (2011) 17:610–7. doi: 10.1038/nm.2353 PMC3270885 21499269

[184] Ghosh AR Bhattacharya R Bhattacharya S Nargis T Rahaman O Duttagupta P. Adipose Recruitment and Activation of Plasmacytoid Dendritic Cells Fuel Metaflammation. Diabetes (2016) 65:3440–52. doi: 10.2337/db16-0331 27561727

[185] Elieh Ali Komi D Shafaghat F Christian M. Crosstalk Between Mast Cells and Adipocytes in Physiologic and Pathologic Conditions. Clin Rev Allergy Immunol (2020) 58:388–400. doi: 10.1007/s12016-020-08785-7 32215785 PMC7244609

[186] Yabut JM Desjardins EM Chan EJ Day EA Leroux JM Wang B. Genetic Deletion of Mast Cell Serotonin Synthesis Prevents the Development of Obesity and Insulin Resistance. Nat Commun (2020) 11:463. doi: 10.1038/s41467-019-14080-7 31974364 PMC6978527

[187] Hubler MJ Peterson KR Hasty AH. Iron Homeostasis: A New Job for Macrophages in Adipose Tissue? Trends Endocrinol Metab (2015) 26:101–9. doi: 10.1016/j.tem.2014.12.005 PMC4315734 25600948

[188] Knights AJ Vohralik EJ Houweling PJ Stout ES Norton LJ Alexopoulos SJ. Eosinophil Function in Adipose Tissue Is Regulated by Krüppel-Like Factor 3 (KLF3). Nat Commun (2020) 11:2922. doi: 10.1038/s41467-020-16758-9 32523103 PMC7286919

[189] Winer S Chan Y Paltser G Truong D Tsui H Bahrami J. Normalization of Obesity-Associated Insulin Resistance Through Immunotherapy: CD4+ T Cells Control Glucose Homeostasis. Nat Med (2009) 15:921–9. doi: 10.1038/nm.2001 PMC3063199 19633657

[190] Medrikova D Sijmonsma TP Sowodniok K Richards DM Delacher M Sticht C. Brown Adipose Tissue Harbors a Distinct Sub-Population of Regulatory T Cells. PloS One (2015) 10:e0118534. doi: 10.1371/journal.pone.0118534 25714366 PMC4340926

[191] Cipolletta D. Adipose Tissue-Resident Regulatory T Cells: Phenotypic Specialization, Functions and Therapeutic Potential. Immunology (2014) 142:517–25. doi: 10.1111/imm.12262 PMC4107662 24484282

[192] Hu B Jin C Zeng X Resch JM Jedrychowski MP Yang Z. γδ T Cells and Adipocyte IL-17RC Control Fat Innervation and Thermogenesis. Nature (2020) 578:610–4. doi: 10.1038/s41586-020-2028-z PMC7055484 32076265

[193] Kohlgruber AC Gal-Oz ST LaMarche NM Shimazaki M Duquette D Nguyen HN. γδ T Cells Producing Interleukin-17A Regulate Adipose Regulatory T Cell Homeostasis and Thermogenesis. Nat Immunol (2018) 19:464. doi: 10.1038/s41590-018-0094-2 29670241 PMC8299914

[194] Lynch L Michelet X Zhang S Brennan PJ Moseman A Lester C. Regulatory iNKT Cells Lack PLZF Expression and Control Treg Cell and Macrophage Homeostasis in Adipose Tissue. Nat Immunol (2015) 16:85–95. doi: 10.1038/ni.3047 25436972 PMC4343194

[195] Lynch L Hogan AE Duquette D Lester C Banks A LeClair K. iNKT Cells Induce FGF21 for Thermogenesis and Are Required for Maximal Weight Loss in GLP1 Therapy. Cell Metab (2016) 24:510–9. doi: 10.1016/j.cmet.2016.08.003 PMC5061124 27593966

[196] Macdougall CE Wood EG Loschko J Scagliotti V Cassidy FC Robinson ME. Visceral Adipose Tissue Immune Homeostasis Is Regulated by the Crosstalk Between Adipocytes and Dendritic Cell Subsets. Cell Metab (2018) 27:588–601.e4. doi: 10.1016/j.cmet.2018.02.007 29514067 PMC5846800

